# Role of K_ATP_ Channels in Glucose-Regulated Glucagon Secretion and Impaired Counterregulation in Type 2 Diabetes

**DOI:** 10.1016/j.cmet.2013.10.014

**Published:** 2013-12-03

**Authors:** Quan Zhang, Reshma Ramracheya, Carolina Lahmann, Andrei Tarasov, Martin Bengtsson, Orit Braha, Matthias Braun, Melissa Brereton, Stephan Collins, Juris Galvanovskis, Alejandro Gonzalez, Lukas N. Groschner, Nils J.G. Rorsman, Albert Salehi, Mary E. Travers, Jonathan N. Walker, Anna L. Gloyn, Fiona Gribble, Paul R.V. Johnson, Frank Reimann, Frances M. Ashcroft, Patrik Rorsman

**Affiliations:** 1 Oxford Centre for Diabetes, Endocrinology, and Metabolism, University of Oxford, Churchill Hospital, Oxford OX3 7LJ, UK; 2 Department of Physiology, Anatomy, and Genetics, Henry Wellcome Centre for Gene Function, University of Oxford, Parks Road, Oxford OX1 3PT, UK; 3 Oxford National Institute for Health Research, Biomedical Research Centre, Churchill Hospital, Oxford OX3 7LJ, UK; 4 Cambridge Institute for Medical Research and Institute of Metabolic Science, Addenbrooke’s Hospital, Hills Road, Cambridge CB2 0XY, UK

## Abstract

Glucagon, secreted by pancreatic islet α cells, is the principal hyperglycemic hormone. In diabetes, glucagon secretion is not suppressed at high glucose, exacerbating the consequences of insufficient insulin secretion, and is inadequate at low glucose, potentially leading to fatal hypoglycemia. The causal mechanisms remain unknown. Here we show that α cell K_ATP_-channel activity is very low under hypoglycemic conditions and that hyperglycemia, via elevated intracellular ATP/ADP, leads to complete inhibition. This produces membrane depolarization and voltage-dependent inactivation of the Na^+^ channels involved in action potential firing that, via reduced action potential height and Ca^2+^ entry, suppresses glucagon secretion. Maneuvers that increase K_ATP_ channel activity, such as metabolic inhibition, mimic the glucagon secretory defects associated with diabetes. Low concentrations of the K_ATP_ channel blocker tolbutamide partially restore glucose-regulated glucagon secretion in islets from type 2 diabetic organ donors. These data suggest that impaired metabolic control of the K_ATP_ channels underlies the defective glucose regulation of glucagon secretion in type 2 diabetes.

## Highlights

• K_ATP_ channel closure stimulates insulin secretion but inhibits glucagon release
• α cell depolarization reduces voltage-gated Ca^2+^ entry and glucagon release
• An activating K_ATP_ channel mutation impairs glucagon release in mice
• K_ATP_ channel closure corrects glucagon secretion defect in type 2 diabetic islets

## Graphical Abstract

**Figure:**
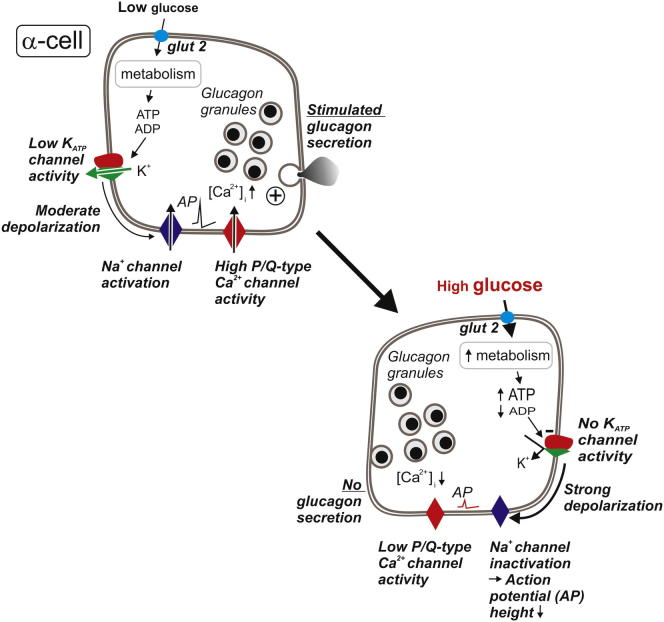

## Introduction

Glucagon and insulin are the body’s principal plasma glucose-regulating hormones. They are secreted from the α and β cells of the pancreatic islets, respectively. Physiologically, glucagon is released in response to a fall in plasma glucose levels, an increase in amino acids, and β-adrenergic stimulation (Gromada et al., 2007). Diabetes is a bihormonal disorder involving both inadequate insulin secretion and defective glucagon secretion. The glucagon secretory defects include oversecretion at high glucose (when it is not needed) and inadequate release at low glucose (when it is needed) (Cryer, 2002; Unger and Cherrington, 2012).

Whereas the cellular regulation of insulin secretion is fairly well understood (Remedi and Nichols, 2009; Seino et al., 2011), much less is known about the control of glucagon secretion (Gaisano et al., 2012). Hypotheses for the regulation of glucagon secretion include paracrine effects, mediated by factors released from neighboring insulin-secreting β cells or somatostatin-secreting δ cells, or innervation (Gromada et al., 2007). However, in both human and rodent islets, glucagon secretion is strongly inhibited by glucose concentrations that have little stimulatory effect on insulin secretion (Walker et al., 2011), and glucose remains capable of suppressing secretion following pharmacological or immunological inhibition of somatostatin signaling (de Heer et al., 2008; Vieira et al., 2007). Moreover, glucagon secretion responds normally to hypoglycemia after denervation of the pancreas (Sherck et al., 2001). These considerations suggest that α cells, in addition to being under paracrine control, possess an intrinsic glucose-sensing mechanism. This remains poorly defined, but studies on K_ATP_-channel knockout mice indicate that K_ATP_-channels are somehow involved (Cheng-Xue et al., 2013; Gromada et al., 2004; Muñoz et al., 2005; Shiota et al., 2005) and the inhibitory effect of high glucose can be reversed by low concentrations of the K_ATP_-channel activator diazoxide (Göpel et al., 2000b; MacDonald et al., 2007). In pancreatic β cells, closure of these channels by metabolically generated ATP leads to membrane depolarization, electrical activity, and insulin secretion. It is not immediately evident, however, how regulation of the same channels by glucose in α cells could suppress glucagon secretion. To date, most studies have failed to detect an effect of glucose on α cell K_ATP_-channel activity (Barg et al., 2000; Bokvist et al., 1999; Quoix et al., 2009; Ramracheya et al., 2010), but one study reported a small glucose-induced decrease in K_ATP_ channel activity that, paradoxically, was associated with stimulation rather than inhibition of glucagon secretion (Olsen et al., 2005). Notably, all these studies of K_ATP_-channel activity used isolated α cells in tissue culture. Tissue culture and/or deprivation of the normal intercellular milieu following islet dissociation may affect α cell function via altered gene transcription, protein expression, or loss of normal paracrine signaling. To avoid such potential confounding effects, the experiments now reported were (whenever technically feasible) performed on α cells in freshly isolated intact islets.

Here we show that glucose-induced inhibition of K_ATP_-channels in α cells results in inhibition of glucagon secretion, that the glucagon secretory defects associated with diabetes can be mimicked by experimental conditions leading to a tiny increase in K_ATP_-channel activity, and that glucose-regulated glucagon secretion can be restored in diabetic or metabolically compromised islets by low concentrations of the K_ATP_-channel blocker tolbutamide.

## Results

### Glucose Regulates Glucagon Secretion by an Intrinsic, Nonparacrine Mechanism

**Figure 1:**
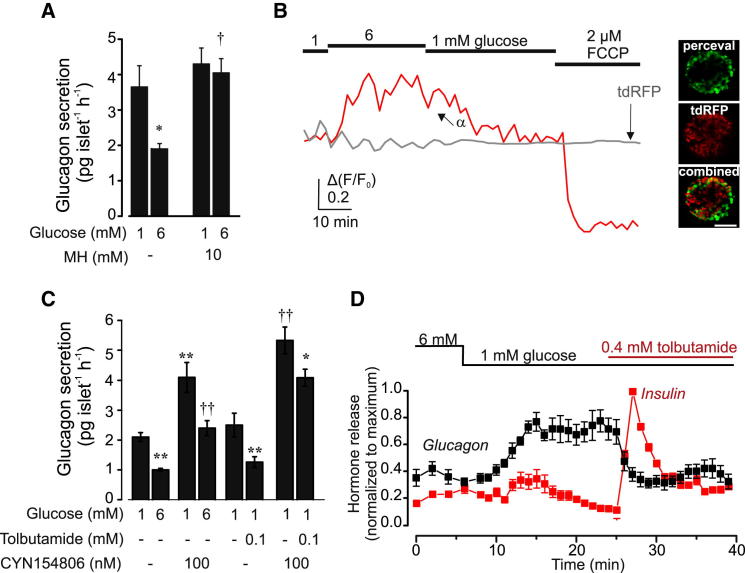
Regulation of Glucagon Secretion by Glucose and Tolbutamide (A) Glucagon secretion from mouse islets at 1 mM and 6 mM glucose in the absence and presence of 10 mM mannoheptulose (MH, n = 6). ^∗^p < 0.05 versus 1 mM glucose; †p < 0.05 versus 6 mM glucose without mannoheptulose. (B) (Left) Representative traces of the increase in [ATP/ADP]_cyt_ measured as Δ(*F*/*F*_0_) recorded from individual α cells within the islet, in response to 6 mM glucose and 2 μM FCCP. The gray line represents parallel measurements of tdRFP fluorescence in the α cell. The data are representative of 44 α cells in 9 intact islets from 3 different animals. (Right) Confocal images of Perceval (green), tdRFP (red), and the combination of the two (yellow). Scale bar, 200 μm. (C) Glucagon secretion from muse islets in the absence or presence of CYN154806 (which blocks SSTR2, the main somatostatin receptor subtype in α cells; Yue et al., 2012) at 1 mM or 6 mM glucose (left) or with and without tolbutamide (0.1 mM, right; n = 6–7). ^∗^p < 0.05 or ^∗∗^p < 0.01 versus 1 mM glucose; ^††^p < 0.01 versus 6 mM glucose without CYN154806. (D) Insulin and glucagon secretion were measured using the perfused pancreas preparation. Data are normalized to the maximal insulin or glucagon secretion in each mouse (n = 5–7). For clarity, significances are not indicated, but effects of glucose and tolbutamide on insulin and glucagon secretion were statistically significant (^∗^p < 0.05 or better). Error bars indicate SEM.

We first established that the inhibitory effect of glucose on glucagon secretion is secondary to glucose metabolism. Mannoheptulose, an inhibitor of glucose phosphorylation, abolished the inhibitory effect of 6 mM glucose on glucagon secretion, without affecting hormone release at 1 mM glucose (Figure 1A).

We used the fluorescent probe Perceval to measure the cytosolic ATP/ADP ratio ([ATP]/[ADP]_cyt_) in α cells of intact mouse islets (Figure 1B). Addition of the mitochondrial uncoupler FCCP promptly decreased [ATP]/[ADP]_cyt._. On average, glucose increased [ATP]/[ADP]_cyt_ (measured as relative increase in Perceval fluorescence above basal) by 33% ± 2% (n = 44; p < 0.02, Mann-Whitney U test for independent samples). In these experiment, α cells were identified by tdRFP fluorescence. We have considered the possibility that tdRFP might interfere with the ATP measurements. However, we do not think this is the case. First, background tdRFP fluorescence was stable and unaffected by glucose or FCCP (Figure 1B). Second, effects of glucose in α cells identified by spontaneous oscillations in [Ca^2+^]_i_ at 1 mM glucose were very similar to those seen in α cells identified by tdRFP fluorescence. Thus, we conclude that glucose increases ATP in α cells. These findings are in agreement with earlier reports of a small (10%–20%) increase in cytoplasmic [ATP] (Ishihara et al., 2003; Ravier and Rutter, 2005) and of total ATP and ADP (Detimary et al., 1998) in response to glucose in α cells using alternative methods of measurement.

Both somatostatin and insulin have been proposed to mediate the inhibitory effects of glucose on glucagon secretion in intact islets (Unger and Cherrington, 2012; Unger and Orci, 2010). The somatostatin receptor subtype 2 (SSTR2) antagonist CYN154806 increased glucagon secretion at both 1 and 6 mM glucose, suggesting α cells are under tonic somatostatin inhibition. In freshly isolated islets, tolbutamide (unlike what was previously observed in cultured islets; Cheng-Xue et al., 2013) remained inhibitory on glucagon secretion when somatostatin signaling was acutely inhibited using CYN154806 (Figure 1C).

Figure 1D shows the reciprocal increase in insulin secretion and decrease in glucagon secretion produced when glucose is lowered from 6 mM to 1 mM. Glucagon secretion was maximally stimulated before any detectable inhibition of insulin secretion. Tolbutamide produced a transient 10-fold stimulation of insulin secretion and mimicked the inhibitory effect of glucose on glucagon secretion, but glucagon secretion remained suppressed even when stimulated insulin release had decayed by 80%. The report that glucagon secretion evoked by insulin-induced hypoglycemia is not very different in control mice and in mice lacking insulin receptors in α cells (Kawamori et al., 2009) provides additional evidence for a dissociation between insulin and glucagon secretion.

Collectively, these data suggest that glucose inhibits glucagon secretion by mechanisms that do not require paracrine effects of insulin or somatostatin (or signals coreleased with these hormones). However, paracrine mechanisms may become functionally more significant under physiological situations associated with strong stimulation of insulin and somatostatin secretion.

### Effects of Glucose and Pharmacological Modulators of K_ATP_ Channel Activity on α Cell Electrical Activity

**Figure 2:**
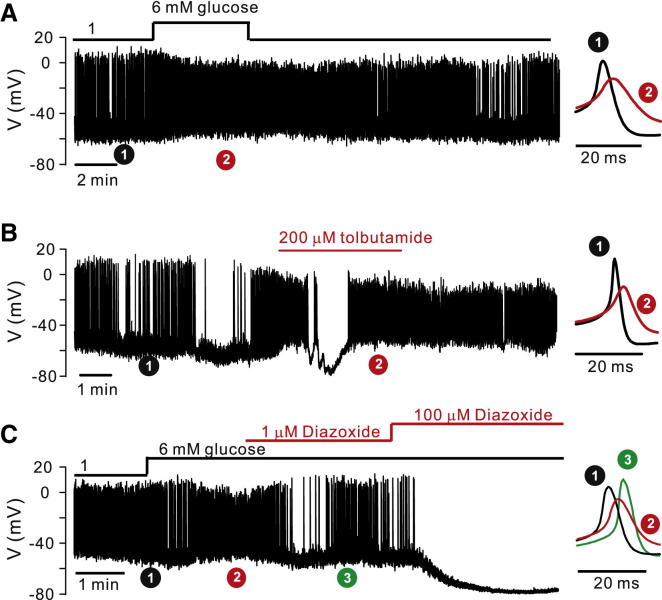
Effects of Glucose, Tolbutamide, and Diazoxide on Electrical Activity in α Cells (A–C) (Left) Electrical activity in mouse α cells (identified by action potential firing at 1 mM glucose) at 1 mM and 6 mM glucose (A, n = 9), 200 μM tolbutamide (B, n = 5), or 1 or 100 μM diazoxide (C, n = 5). (Right) Averaged action potentials recorded around the indicated time points, shown on an expanded timescale (averages of 308 and 181 spikes at 1 and 6 mM glucose in A; 54 and 223 in the absence and presence of tolbutamide in B; and 277, 560, and 190 at 1 mM and 6 mM glucose, and 6mM glucose + 1 μM diazoxide in C). See also Figures S1 and S2 and Table S1.

Unlike insulin-secreting β cells and somatostatin-secreting δ cells (Göpel et al., 2000a), pancreatic α cells in intact mouse islets exposed to 1 mM glucose (i.e., when glucagon secretion is stimulated) generate spontaneous action potentials (Figure 2A). The frequency, the peak voltage, and the most negative membrane potential attained during action potential firing averaged 1.4 ± 0.3 Hz, 2 ± 3 mV, and −54 ± 2 mV (n = 9). Corresponding values in the presence of 6 mM glucose (when glucagon secretion is maximally suppressed) were 3.0 ± 0.5 Hz (p < 0.02), −7 ± 2 mV (p < 0.002), and −45 ± 2 mV (p < 0.01; n = 9). Averaged action potentials (see Experimental Procedures) recorded under the indicated experimental conditions are shown on the right on an expanded time base.

Tolbutamide mimicked the effect of elevating glucose (Figure 2B). It depolarized α cells by 11 ± 3 mV (p < 0.02), reduced action potential peak voltage by 17 ± 6 mV (p < 0.02), and increased firing frequency to 3.3 ± 0.7 Hz (p < 0.02; n = 5).

The effect of the K_ATP_-channel activator diazoxide on α cells exposed to 6 mM glucose was concentration dependent (Figure 2C). At 100 μM, it hyperpolarized α cells to −74 ± 4 mV (p < 0.01; n = 4) and abolished action potential firing. At 1 μM it hyperpolarized α cells by 9 ± 2 mV (p < 0.01) yet *increased* action potential peak voltage by 7 ± 2 mV (p < 0.02, n = 5).

The maximum rate of depolarization (d*V*/d*t*_*d*_) during the upstroke of the action potential averaged 37 ± 6 V/s (n = 11) at 1 mM glucose, 15 ± 3 V/s (n = 11; p < 0.001 versus 1 mM glucose) at 6 mM glucose (or 0.2 mM tolbutamide), and 33 ± V/s (n = 5; p < 0.02 versus 6 mM glucose) in the presence of 6 mM glucose and 1 μM diazoxide. Corresponding values for the maximum rate of repolarization (d*V*/d*t*_r_) averaged −24 ± 3 V/s, −13 ± 1 V/s (p < 0.01 versus 1 mM glucose), and −31 ± 6 V/s (p < 0.05 versus 6 mM glucose). We attribute the slower repolarization in the presence of glucose or tolbutamide to reduced voltage-dependent activation of the K^+^-channels involved in action potential repolarization when spike height is reduced.

In a small number of experiments (4 of 29 cells), elevating glucose transiently hyperpolarized the α cell and suppressed action potential firing (Figure S1A available online). This could be antagonized by CYN154806, suggesting it may result from somatostatin released by neighboring δ cells (Figure S1B).

Some α cells hyperpolarized spontaneously in the continued presence of 1 mM glucose (Figure S1C). This repolarization and suppression of electrical activity lasted ∼5 min, after which the α cell depolarized and action potential firing resumed. Spontaneous membrane potential oscillations were seen in α cells exposed to 1 mM glucose in 60% of experiments. These probably account for the [Ca^2+^]_I_ oscillations with a period of ∼5 min seen in isolated islets at low glucose (Figure S1D). The frequency and amplitude of these oscillations were little affected by 6 mM glucose, as previously reported (Le Marchand and Piston, 2010).

### Effects of Glucose and Tolbutamide on Whole-Cell K_ATP_ Channel Activity

**Figure 3:**
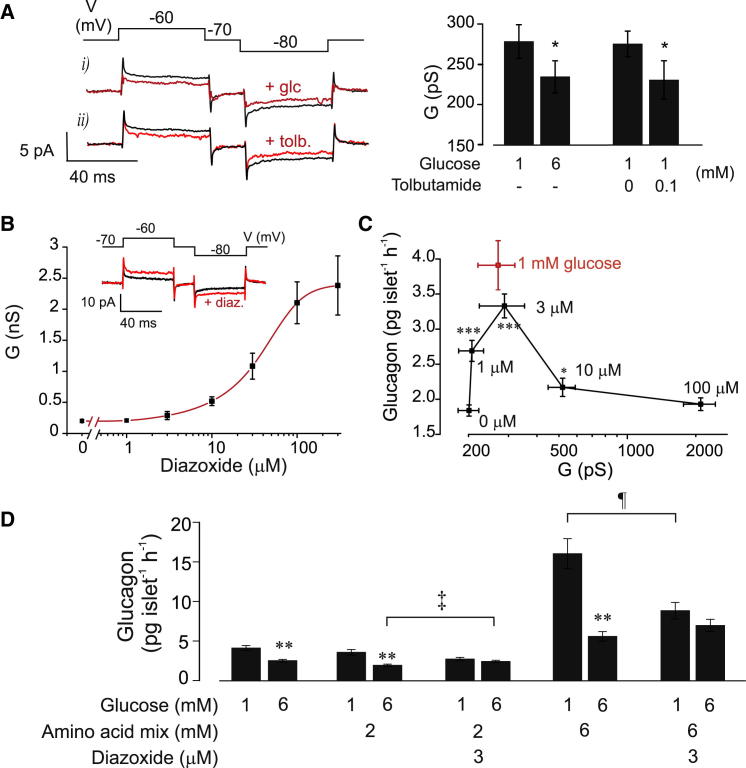
Modulation of K_ATP_ Channel Activity by Glucose in Mouse Pancreatic α Cells (A) (Left) Perforated patch whole-cell currents in mouse α cells evoked by ±10 mV voltage pulses (indicated schematically above the current traces) at 1 mM glucose (black) and 6 mM glucose (red, +glc.) or 1 mM glucose plus 100 μM tolbutamide (red, +tolb.). Histograms (right) show mean whole-cell K_ATP_-currents (expressed as membrane conductance) in glucose and tolbutamide, as indicated. ^∗^p < 0.05 versus 1 mM glucose. (B) Dose-response curve for the effects of increasing concentrations of diazoxide (1–300 μM) on the whole-cell K_ATP_-channel activity (expressed as membrane conductance) in α cells within intact islets. ^∗^p < 0.05 versus no diazoxide for concentrations >1 μM (not indicated for clarity). (Inset) Perforated patch whole-cell currents in mouse α cells evoked by ±10 mV voltage pulses at 6 mM glucose (black) and 6 mM glucose + 3 μM diazoxide (red, +diaz.). (C) Relationship between glucagon secretion and mean whole-cell conductance (*G,* perforated patch) in mouse α cells at 6 mM glucose and the indicated diazoxide concentrations (black). Red square, glucagon secretion and *G* at 1 mM glucose. ^∗∗∗^p < 0.001 versus no diazoxide. (D) Effects of glucose and diazoxide on glucagon secretion in the absence and presence of 2–6 mM of amino acid mixtures (see Table S1). Glucose and diazoxide were also added to the medium, as indicated. n = 8–9 experiments. ^∗∗^p < 0.01 for effects of 1 mM glucose versus 6 mM glucose (at 0, 2, or 6 mM AAM); ‡p < 0.05 for effect of diazoxide versus no diazoxide at 6 mM glucose in the presence of 2 mM AAM; ¶p < 0.05 for effect of diazoxide at 1 mM glucose in the presence of 6 mM AAM. Error bars indicate SEM. See also Figure S2 and Table S1.

Figures 2A–2C suggest that, at 1 mM glucose, α cells exhibit low K_ATP_-channel activity and that glucose acts by modulating this activity. We therefore recorded whole-cell K_ATP_-current under voltage-clamp conditions in identified α cells (Figures S2C and S2D) in intact islets (Figure 3A). Data are expressed as changes in whole-cell conductance (*G*). At 1 mM glucose, *G* was 270 ± 50 pS (n = 16). Increasing glucose to 6 mM, or addition of tolbutamide, reduced *G* by 75 ± 39 pS (p < 0.05; n = 16) and 76 ± 32 pS (p < 0.01; n = 14), respectively. Tolbutamide was ineffective at 6 mM glucose (data not shown), as expected if glucose had already closed K_ATP_-channels. The glucose- and tolbutamide-induced changes in *G* were small—less than 3% of those seen in β cells under comparable conditions (Zhang et al., 2008). However, intracellular application of 0.1 mM ADP (to maximally activate K_ATP_-channels) increased *G* from an initial value of 0.30 ± 0.03 nS to 11 ± 2 nS (n = 5; Figures S2A and S2B). Thus, the K_ATP_-channel is under strong tonic inhibition (>99%) in α cells. The reported >5-fold greater ATP sensitivity of K_ATP_-channel activity in α cells than in β cells (Leung et al., 2005) might contribute to the strong tonic inhibition of K_ATP_-channels in α cells.

### Relationship between K_ATP_ Channel Activity and Glucagon Secretion

Diazoxide activated K_ATP_-channels inhibited by glucose, with half-maximal stimulation occurring at 29 ± 6 μM (n = 5; Figure 3B). Glucagon secretion was measured in the presence of 6 mM glucose (to maximally inhibit secretion) and increasing concentrations of diazoxide. This enables the relationship between K_ATP_ conductance (*G*, determined as above) and glucagon secretion to be determined over a wider range of conductance than can be obtained by varying glucose concentration (Figure 3C). The *G*-secretion relationship was bell shaped, being maximal at ∼280 pS (3 μM diazoxide), where both *G* and secretion were similar to that at 1 mM glucose. Importantly, both small (∼100 pS) increases and decreases in *G* inhibited glucagon secretion. The ability of diazoxide to reverse the effects of glucose cannot be explained by relief from paracrine suppression of glucagon secretion by insulin or somatostatin, as glucose-induced release of these hormones is unaffected by diazoxide concentrations as high as 30 μM and 200 μM, respectively (MacDonald et al., 2007; Zhang et al., 2007).

The data of Figure 3C confirm our earlier finding (MacDonald et al., 2007), that low concentrations of diazoxide antagonize the inhibitory effect of glucose on glucagon secretion. We also tested the effects of glucose and diazoxide in the presence of two different amino acid mixtures, AAMa (2 mM) and AAMb (6 mM), designed to simulate the conditions occurring during fasting (Rudman et al., 1989) and following a protein-rich meal, respectively (Figure 3D). AAMa did not detectably enhance glucagon secretion beyond that evoked by 1 mM glucose. Increasing glucose to 6 mM in the presence of AAMa inhibited glucagon secretion as strongly as it did under control conditions, an effect that was prevented by diazoxide. In fact, diazoxide *stimulated* glucagon secretion in the presence of 6 mM glucose. AAMb stimulated glucagon secretion ∼4-fold at 1 mM glucose and elevation of glucose to 6 mM reduced glucagon secretion by >60%. Again, no inhibitory effect of glucose was observed in the presence of 3 μM diazoxide. These observations argue that K_ATP_-channel closure plays a key role in the modulation of glucagon secretion by glucose in both the absence and presence of amino acids.

### Membrane Potential-Dependent Reduction of Action Potential Height

**Figure 4:**
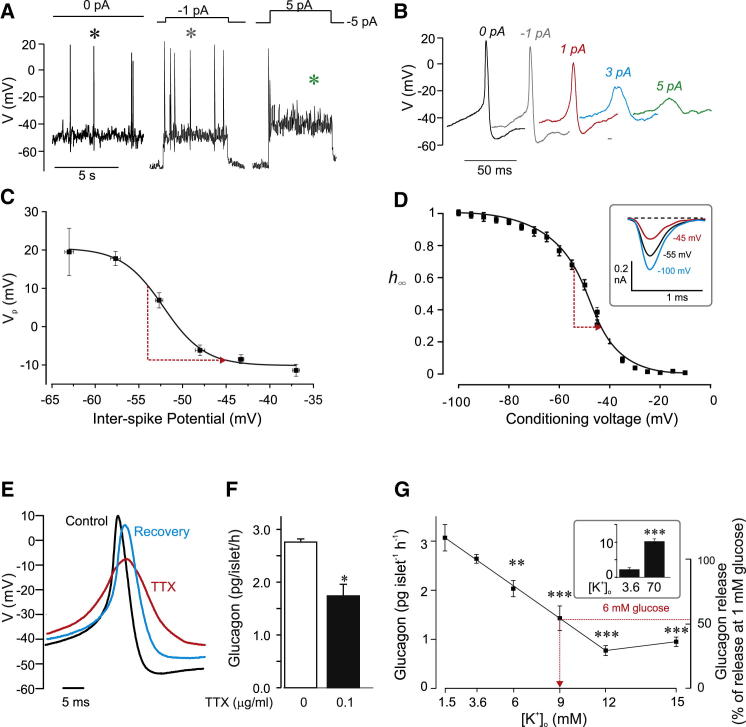
Impact of Membrane Potential on Action Potential Amplitude (A) Electrical activity in mouse α cells hyperpolarized to ∼−80 mV by injection of −5 pA and stimulated by 5 s current pulses to different membrane potentials applied at a frequency of 0.2 Hz. (B) Individual spikes from (A), as indicated by asterisks, shown on an expanded timescale. (C) Relationship between interspike membrane potential and action potential peak voltage (*V*_p_). The amplitude of each action potential and the associated interspike voltage were measured. For display, data were binned according to the most negative interspike potential (bin width, 5 mV) and averaged (n = 7–12 action potentials for each data point). The line is a Boltzmann fit to the mean data with a midpoint of −52 mV. The red arrow indicates the decrease in peak voltage predicted from the 9 mV glucose-induced depolarization. (D) Voltage-dependent inactivation of Na^+^-current in identified α cells in intact islets. Peak currents during a 1 ms test pulses to 0 mV are displayed against the membrane potential during the preceding 200 ms conditioning pulses (frequency, 1 Hz). Current responses are presented as h_∞_ (= *I*/*I*_max_). The response following a conditioning pulse to −100 mV was taken as unity (n = 6). The midpoint of inactivation was −47 mV. Arrow indicates the decrease in h_∞_ predicted from the glucose-induced depolarization. (Inset) Na^+^-currents during depolarizations to 0 mV following conditioning pulses to −100 mV, −55 mV, and −45 mV. See also Table S1. (E) Examples of action potentials recorded from an α cell in an intact islet exposed to 1 mM glucose under control conditions, after addition of TTX (0.1 μg/ml) and following washout of the blocker (ten individual spikes averaged for each experimental condition). (F) Effects of TTX on glucagon secretion measured from isolated NMRI mouse islets at 1 mM glucose in the absence and presence of TTX (as indicated). ^∗^p < 0.05 versus 1 mM glucose without TTX. (G) Absolute (left) and relative (right) glucagon secretion from mouse islets at 1 mM glucose and different [K^+^]_o_ (n = 6). Relative release is normalized to that at 3.6 mM [K^+^]_o_). The red arrow indicates the equivalent decrease (47% ± 4%) in glucagon secretion produced by increasing glucose from 1 mM to 6 mM in control experiments (at 3.6 mM [K]_o_; seven different experimental series performed over several years). (Inset) Stimulation of glucagon secretion by 70 mM [K^+^]_o_. ^∗^p < 0.01 and ^∗∗∗^p < 0.001 versus 3.6 mM [K^+^]_o_. Error bars indicate SEM.

Figures 2 and 3 suggest K_ATP_-channel closure leads to membrane depolarization and reduced action potential amplitude. We investigated the relationship between interspike membrane potential and the voltage at the peak of the action potential (Figures 4A and 4B). Peak voltage *decreased* with interspike depolarization, from +18 ± 6 mV at negative membrane potentials (below −60 mV) to −14 ± 2 mV at depolarized potentials (above −40 mV), being half-maximal at −52 ± 3 mV with a slope factor of 4 ± 1 mV (n = 4 cells from four different islets and three different mice; Figure 4C). Using these values, we estimate that the 9 mV depolarization produced by increasing glucose from 1 mM to 6 mM will reduce action potential peak voltage from +6 mV to −9 mV, in reasonable agreement with values observed experimentally (Figure 2A). Thus, the glucose-induced changes in membrane potential are sufficient to account for the reduction of action potential height.

Action potential firing in α cells depends on the opening of voltage-gated Na^+^-channels (Göpel et al., 2000b). We reasoned that glucose-induced membrane depolarization might reduce action potential height by voltage-dependent inactivation of Na^+^-channels. In agreement with this idea, the Na^+^-channel blocker TTX (0.1 μg/ml) reduced action potential peak voltage in α cells exposed to 1 mM glucose by 14 ± 4 mV (n = 5; p < 0.01; Figure 4E), reduced d*V*/d*t*_*d*_ from 37 ± 12 V/s to 11 ± 2 V/s (p < 0.05 versus no TTX), and reduced d*V*/d*t*_*r*_ from −22 ± 4 V/s to −13 ± 3 V/s (p < 0.05 versus no TTX). It also inhibited glucagon secretion at 1 mM glucose by ∼40% (Figure 4F). The peak Na^+^-current amplitude in identified α cells decreased steeply at potentials positive to −60 mV (Figure 4F): the relationship between membrane potential and peak Na^+^-current predicts that the 9 mV depolarization produced by glucose will reduce the fraction of active Na^+^-channels (h_∞_) from 0.7 to 0.3 (red arrow). These data argue that glucose-induced membrane depolarization mediates its effect on action potential height via (partial) voltage-dependent inactivation of voltage-gated Na^+^-channels.

We next explored the effect of membrane potential on glucagon secretion, by varying the extracellular K^+^ concentration ([K^+^]_o_) (Figure 4G). In the presence of 1 mM glucose, elevation of [K]^+^_o_ from 1.5 mM to 12 mM progressively *decreased* glucagon secretion and thus mimicked the effect of high glucose. High [K^+^]_o_ (70 mM) stimulated glucagon secretion >4-fold (inset). From the relationship between [K^+^]_o_ and glucagon secretion we estimate that elevating [K^+^]_o_ from 3.6 mM to 9 mM is required to produce the same inhibition of glucagon secretion as that resulting from an increase in glucose from 1 mM to 6 mM (arrow). This increase in [K^+^]_o_ corresponds to an estimated 13 mV depolarization (estimated from the relationship between [K^+^]_o_ and α cell membrane potential reported previously; De Marinis et al., 2010), which is in reasonable agreement with the 9 mV glucose-induced depolarization observed experimentally (Figure 2A).

### Glucose Inhibits Glucagon Secretion by Reducing P/Q-Type Ca^2+^ Channel Activation

**Figure 5:**
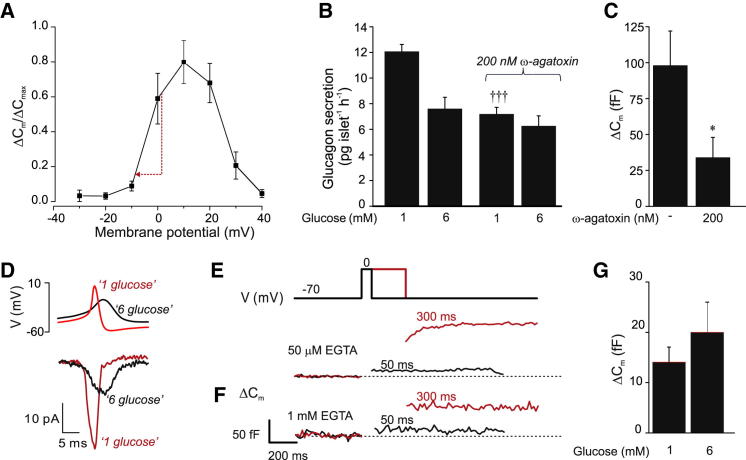
Ca^2+^ Channel Activation and Exocytosis in α Cells (A) Voltage dependence of α cell exocytosis (n = 5) was measured as changes in membrane capacitance (ΔC_m_) in response to 500 ms voltage pulses (holding potential −70 mV) using the standard whole-cell configuration. ΔC_m_ was normalized to the largest response in each cell (ΔC_max_). The red arrow indicates the 75% suppression of exocytosis predicted to result from a 9 mV reduction of the action potential. (B) Glucagon secretion from islets exposed to glucose and ω-agatoxin, as indicated (n = 6). ^∗∗^p < 0.01 versus 1 mM glucose under control conditions; ^†††^p < 0.001 versus 1 mM glucose under control conditions. (C) ΔC_m_ recorded during 500 ms depolarizations to 0 mV in α cells with/without 200 nM ω-agatoxin (n = 4). ^∗^p < 0.05 versus no ω-agatoxin. (D) ω-agatoxin-sensitive Ca^2+^-currents (lower) evoked by voltage-clamp commands based on the averages of 20 action potentials recorded in the presence of 1 and 6 mM glucose (top). (E and F) ΔC_m_ recorded during 50 and 300 ms depolarizations to 0 mV with intracellular EGTA included in the pipette medium at 50 μM (E) or 1 mM (F). (G) ΔC_m_ recorded in the perforated patch whole-cell configuration at 1 and 6 mM glucose in response to 500 ms voltage pulses from −70 mV to 0 mV (n = 10). Error bars indicate SEM. See also Figure S3 and Table S1.

Figure 5A summarizes the voltage dependence of exocytosis in identified α cells in intact islets. The relationship predicts that the 9 mV reduction in spike height produced by glucose reduces exocytosis by 75%.

Glucagon exocytosis is dependent on Ca^2+^ entry via voltage-gated Ca^2+^-channels (see Figure S3). The P/Q type Ca^2+^-channel blocker ω-agatoxin mimicked the inhibitory effect of 6 mM glucose (Figure 5B) and inhibited depolarization-evoked exocytosis (Figure 5C). The reduction in action potential height produced by 6 mM glucose resulted in 66% ± 10% (n = 5; p < 0.01) inhibition of the peak P/Q type Ca^2+^-current evoked by simulated action potentials (Figure 5D). During brief depolarizations (≤50 ms), exocytosis proceeded only during the depolarization (Figure 5E), and not subsequent to it (cf. Zhang et al., 2007). This suggests exocytosis in α cells is controlled by rapid, local [Ca^2+^]_i_ increases, close to the Ca^2+^-channel, and thus echoes Ca^2+^-channel activity. Accordingly, exocytosis elicited by short depolarizations was hardly affected by inclusion of the Ca^2+^-chelator EGTA (1 mM) in the intracellular medium (Figure 5F). On average, 50 ms depolarizations evoked capacitance increases of 14 ± 6 fF (n = 8) and 11 ± 2 fF (n = 5) in the presence of 50 μM and 1 mM intracellular EGTA, respectively. However, exocytosis evoked by 300 ms depolarizations was reduced from 80 ± 20 fF to 30 ± 11 fF (p < 0.05), suggesting that Ca^2+^ diffusion during longer depolarizations may trigger exocytosis of granules not situated in the immediate vicinity of the Ca^2+^-channels.

Glucose (6 mM) did not inhibit α cell exocytosis evoked by membrane depolarization to 0 mV in perforated patch recordings (Figure 5G). This indicates that the glucose-induced suppression of glucagon secretion is secondary to changes in membrane potential rather than a direct effect of the sugar on exocytosis.

### Effects of K_ATP_ Channel Mutations Associated with Reduced ATP Sensitivity on Glucagon Secretion

An increase in K_ATP_-channel activity either stimulates or inhibits glucagon secretion, depending on the magnitude of the increase in the K_ATP_ current (Figure 3C). The question then arises as to whether gain-of-function mutations found in patients with neonatal diabetes (Gloyn et al., 2004) affect glucagon secretion. We addressed this by generating mice expressing K_ATP_-channels with reduced ATP-sensitivity in α cells (α-V59M mice). These had normal body weight (data not shown) and fasting blood glucose levels but exhibited mild glucose intolerance (Figures S4A and S4B) and increased insulin sensitivity (Figure S4C).

**Figure 6:**
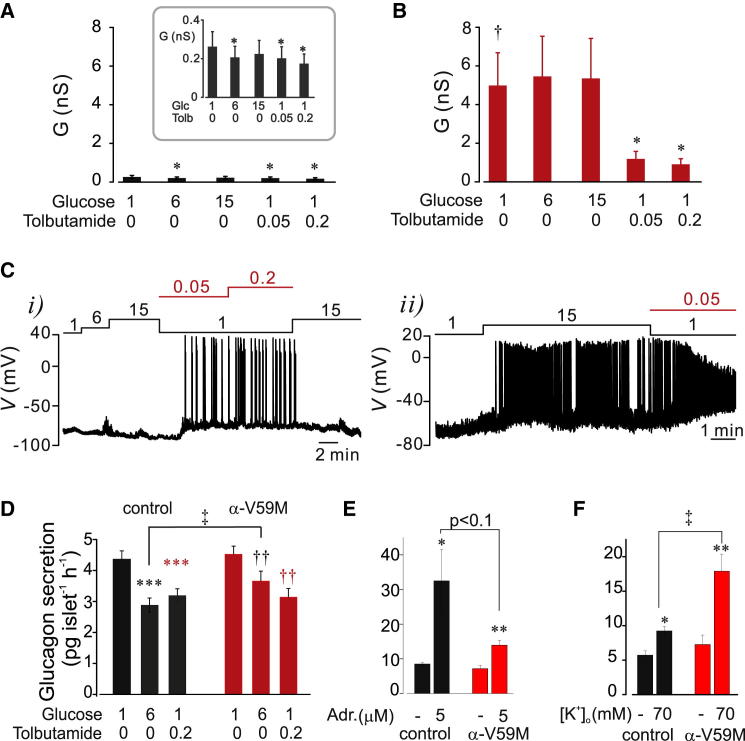
Effects of the Kir6.2-V59M Mutation on Glucagon Secretion (A and B) Whole-cell resting conductance (*G*_r_) measured with the perforated patch method in α cells within control (A; n = 6–7) and α-V59M islets (B; n = 6) at the indicated concentrations (mM) of glucose and tolbutamide. ^∗^p < 0.05 versus 1 mM glucose. †p < 0.05 versus 1 mM glucose in control α cells. Inset in (A) shows conductance changes on an expanded vertical axis. (C) Electrical activity in α cells in α-V59M islets at the indicated glucose (black bar) and tolbutamide (red bar) concentrations. Examples showing α cells (i) refractory to glucose but responding to tolbutamide and (ii) stimulated by glucose and where tolbutamide reduced spike height. (D) Glucagon secretion from control (left and black; n = 15–21 from 8 mice) and α-V59M islets (right and red; n = 26 from 12 mice) at the indicated glucose and tolbutamide concentrations (in mM). ^∗∗∗^p < 0.001 versus 1 mM glucose (control islets), ^††^p < 0.01 versus 1 mM glucose (α-V59M islets), and ^‡^p < 0.05 versus 6 mM glucose in control islets. (E) Effects of adrenaline (adr.; 5 μM) on glucagon secretion at 1 mM glucose in wild-type type (left; n = 6) and α-V59M islets (right; n = 9). ^∗^p < 0.05 and ^∗∗^p < 0.01 versus 1 mM glucose (for comparisons within strain). (F) As in (E), but testing the effects of membrane depolarization produced by increasing extracellular K^+^ to 70 mM. ^∗^p < 0.05 and ^∗∗^p < 0.01 versus 3.6 mM [K^+^]_o_ (for comparisons within strain) and ^‡^p < 0.05 versus 70 mM [K^+^]_o_ in control islets. Error bars indicate SEM. See also Figure S4 and Table S1.

In 1 mM glucose, the membrane conductance (*G*) was >13-fold larger in metabolically intact α cells in islets isolated from α-V59M mice than in control islets (∼1.5 nS/pF versus 0.11 nS/pF; p < 0.03; α cells identified by expression of tdRFP). Whereas 6 mM glucose reduced *G* in control α cells by 17% ± 5%, no reduction was detected in α-V59M α cells (Figure 6A). Tolbutamide reduced *G* by >50% in mutant α cells (Figure 6B), but it still remained >4-fold higher than in control α cells.

We next measured α cell electrical activity in intact control and α-V59M islets. In contrast to control cells (which resemble wild-type cells), only 20% (n = 10) of α-V59M α cells were electrically active in 1 mM glucose. The other 80% were inactive and hyperpolarized (−79 ± 3 mV). When glucose was elevated to 15 mM, two cells depolarized and generated electrical activity, five were refractory to glucose but responded to tolbutamide, and one did not respond to either agent (Figure 6C).

Glucagon content was similar in wild-type and α-V59M islets, averaging 1,589 ± 213 pg/islet and 2,093 ± 207 pg/islet, respectively. Unexpectedly, although 80% of α-V59M α cells were electrically silent at 1 mM glucose, glucagon secretion from α-V59M islets was not less than from control islets (Figure 6D). Notably, the inhibitory effect of glucose was halved in α-V59M islets (−18% versus −35%) whereas tolbutamide was as inhibitory as in control islets (∼30%). In control islets, adrenaline (5 μM) stimulated glucagon secretion ∼4-fold, but this effect was strongly reduced in α-V59M islets (Figure 6E); the adrenaline-induced stimulation averaged 7 ± 1 and 24 ± 4 pg/islet/hr (p < 0.05), respectively. By contrast, glucagon secretion evoked by 70 mM K^+^ was 2.9-fold *larger* in α-V59M than in wild-type islets (Figure 6F).

### A Small Increase in K_ATP_ Channel Activity Mimics the Effects of Type 2 Diabetes on Glucagon Secretion

**Figure 7:**
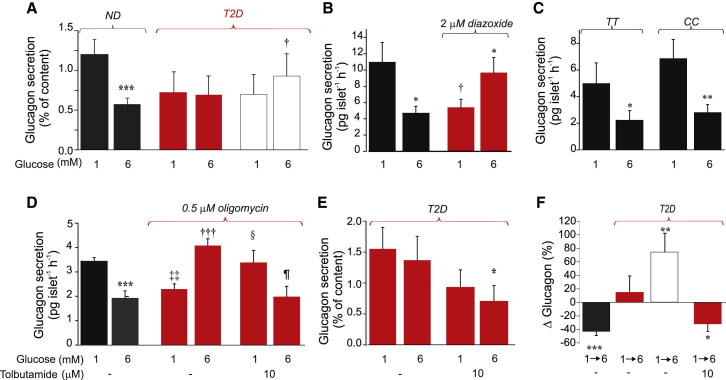
Glucagon Secretion and Type 2 Diabetes (A) Glucagon secretion at 1 and 6 mM glucose in islets from human organ donors without (ND) and with known T2D normalized to glucagon content. White bars show glucagon secretion in the five islet preparations where glucose stimulated glucagon release. ^∗∗∗^p < 0.001 versus 6 mM glucose; ^†^p < 0.02 versus 1 mM glucose in five T2D preparations with no glucose-induced inhibition of glucagon secretion. (B) Glucagon secretion in the absence (black) and presence (red) of 2 μM diazoxide in ND human islets (n = 7 experiments, using islets from two donors). ^∗^p < 0.05 versus 1 mM glucose. ^†^p < 0.05 versus 1 mM glucose in the presence of diazoxide. (C) Glucagon secretion from islets of nondiabetic human donors homozygous for the high-risk (TT; n = 6) and low-risk (CC; n = 8) Kir6.2 (*KCNJ11*) alleles. ^∗^p < 0.05 and ^∗∗^p < 0.01 versus 1 mM glucose. (D) Glucagon secretion from mouse islets at 1 mM and 6 mM glucose in the absence (black) and presence (red) of 0.5 μM oligomycin (n = 8–16). Tolbutamide 10 μM was added as indicated. ^∗∗∗^p < 0.001 versus 1 mM glucose; ^†††^p < 0.001 versus 1 mM glucose plus oligomycin; ^‡‡^p < 0.01 versus 1 mM glucose without oligomycin; ^§^p < 0.05 versus 1 mM glucose with oligomycin (no tolbutamide); ^¶^p < 0.05 versus 1 mM glucose plus oligomycin (with tolbutamide). (E) Effects of glucose (1 or 6 mM) on glucagon secretion normalized to glucagon content in islets from T2D donors in the absence (left) and presence (right; n = 4) of tolbutamide (10 μM). ^∗^p < 0.05 versus 1 mM glucose. (F) Relative effect (%) of elevating glucose from 1 to 6 mM on glucagon secretion in ND (black) and T2D islets (color coded as in A) and in four T2D preparations in the presence of tolbutamide (far right). Significance between 1 mM and 6 mM glucose is indicated. ^∗∗∗^p < 0.001, ^∗∗^p < 0.02, ^∗^p < 0.05. Error bars indicate SEM. See also Figures S5 and S6 and Table S1.

Type 2 diabetes (T2D) is associated with loss of glucose-induced suppression of glucagon secretion—indeed, stimulation may occur instead (Dunning et al., 2005). We evaluated if this disturbance is intrinsic to the islet, using islets isolated from T2D and nondiabetic (ND) organ donors (Figure 7A). In ND islets, glucose (6 mM) inhibited glucagon secretion by ∼50%. No inhibition by glucose was seen in T2D islets. In fact, glucose stimulated glucagon secretion in 5 of the 10 T2D preparations tested but in only 4 of 46 ND preparations (p < 0.001 by χ^2^). Glucagon content was 2.5-fold higher in T2D islets than in ND islets (2,457 ± 461 pg/islet versus 985 ± 105 pg/islet; p < 0.01). The relative effect of glucose on glucagon secretion in ND and T2D islets is summarized in Figure 7F. On average, glucose inhibited glucagon secretion by 43% ± 6% in ND islets but tended to stimulate glucagon secretion by 15% ± 24% in T2D islets. In the five preparations where glucose had the least inhibitory effect, glucose enhanced glucagon secretion by 74% ± 28%. In the remaining five preparations, glucose inhibited glucagon secretion by 44% ± 9% (data not shown).

The inverted response to glucose seen in 50% of T2D islet preparations could be induced in ND islets by 2 μM diazoxide (Figure 7B), which increases K_ATP_-channel activity by ∼80 pS (Figure 3B). In the presence of diazoxide, 6 mM glucose *stimulated* rather than inhibited glucagon secretion. Similar results were observed in mouse islets (data not shown).

A common variant (E23K; rs5219) in *KCNJ11* (which encodes the Kir6.2 subunit of the K_ATP_-channel) is associated with enhanced T2D risk (Gloyn et al., 2003), increased K_ATP_-channel activity (Schwanstecher et al., 2002), and impaired glucose-induced suppression of glucagon secretion in vivo (Tschritter et al., 2002). However, we found no difference in the inhibitory effect of glucose on glucagon secretion in vitro in ND human islets homozygous for the high-risk TT or low-risk CC variants (Figure 7C).

Treatment of ND mouse islets with oligomycin, a blocker of the mitochondrial ATP synthase, converted the inhibitory effect of glucose on glucagon secretion into stimulation (Figure 7D). The ability of glucose to stimulate (rather than inhibit) glucagon secretion in islets treated with oligomycin suggests a regulatory role of nonmitochondrial metabolism in α cells. Interestingly, tolbutamide (10 μM) restored normal glucose regulation of glucagon release from oligomycin-treated islets (Figure 7D) without correcting insulin secretion (data not shown). Collectively, these data suggest that in 50% of T2D patients, possibly because of a metabolic disturbance, K_ATP_-channel activity is slightly increased with resultant loss of glucose-induced suppression of glucagon secretion. If this hypothesis is correct, then it should be possible to reverse the glucagon secretion defect in T2D with tolbutamide. Indeed, as shown in Figures 7E and 7F, tolbutamide (10 μM) partially restored glucose inhibition of glucagon secretion in islets from four T2D organ donors.

## Discussion

Our data show that pancreatic α cells respond to glucose with closure of K_ATP_-channels and suggest that glucose modulates K_ATP_-channel activity and glucagon secretion via increased cytoplasmic ATP/ADP (Figure 1B). Although we favor the idea that α cells possess an intrinsic glucose sensing mechanism, we cannot rule out the involvement of the β cell metabolite GHB (γ-hydroxybutyrate), recently proposed to mediate the inhibitory effect on glucagon secretion (Li et al., 2013). The mechanism by which activation of the GHB receptor would inhibit glucagon secretion has not been established, but our data suggest it may culminate in K_ATP_-channel closure.

### Role of K_ATP_ Channels in Regulating α Cell Excitability

In most cells, K^+^-channel activity dampens cellular excitability by producing membrane hyperpolarization. Conversely, closure of K^+^-channels promotes electrical excitability. The β cell conforms to this principle: K_ATP_ channel closure causes membrane depolarization, electrical activity, and insulin secretion. Strikingly, in α cells, K_ATP_ channel closure also causes depolarization but paradoxically inhibits glucagon secretion. Figure S5 illustrates how our data suggest this difference between α and β cells arises.

Crucially, in α cells, net K_ATP_-channel activity at 1 mM glucose (50 pS; Figure 3A) is only 0.5%–1.5% of the 3–9 nS measured under comparable conditions in β cells (Göpel et al., 2000a; Zhang et al., 2008). As a result, α cells are electrically active at low glucose, stimulating glucagon secretion. Glucose elevation inhibits remaining K_ATP_ channel activity, producing a further depolarization that partially inactivates voltage-dependent Na^+^-channels and decreases spike height. This reduces P/Q-type Ca^2+^-current activation and glucagon exocytosis. Although glucose also increases action potential frequency (+200%; Figure 2), this is insufficient to compensate for the reduced exocytosis (−75%; Figure 5A). The combination of these two effects can be estimated to result in ∼50% inhibition of glucagon secretion, in good agreement with that observed experimentally (Figures 1A, 1C, and 1D).

The much larger K_ATP_ conductance at 1 mM glucose keeps β cells hyperpolarized and electrically silent at low glucose, so that no insulin is secreted. Glucose closes K_ATP_-channels and thereby triggers depolarization, electrical activity, and insulin secretion from a very low basal level. As in α cells, there is also a time-dependent reduction in spike height, but insulin secretion will remain much greater than in 1 mM glucose even after the reduction of spike height has occurred.

Our data suggest that tolbutamide and glucose affect electrical activity and glucagon secretion in freshly isolated islets by identical mechanisms. Very recently, glucose was postulated to inhibit glucagon secretion by a K_ATP_-channel-independent (as yet unidentified) mechanism (Cheng-Xue et al., 2013), but the observation that the glucose-sensitive component of glucagon secretion was reduced by >85% in islets lacking K_ATP_-channels argues that this mechanism is of relatively minor significance.

### Resting Activity of K_ATP_ Channels Determines Islet Hormone Release

The model outlined above postulates that the K_ATP_-channel plays a dual role in the regulation of insulin and glucagon secretion. Depending on the initial activity, channel closure may either stimulate (β cells) or inhibit (α cells) secretion. Thus, increasing basal K_ATP_ activity in α cells may result in a β cell phenotype—i.e., no electrical activity or glucagon secretion at 1 mM glucose, and stimulation of both by glucose. This is precisely what is seen in the presence of low concentrations of diazoxide (Figure 7B). Similarly, we observed that glucose stimulates electrical activity in some α-V59M α cells (Figure 6C). Figure S6 illustrates the relationship between K_ATP_-channel activity and glucagon secretion. Cells with little or no expression of mutant channels behave like control cells (Figure S6A). Cells expressing high levels of mutant channels (or exposed to high concentrations of diazoxide) will be permanently hyperpolarized and show no glucagon secretion at either 1 or 6 mM glucose (Figure S6B). Cells lying between these extremes will show a spectrum of responses: in some, 6 mM glucose will stimulate glucagon secretion (Figure S6C); in others, glucagon release will be high and unaffected by glucose (Figure S6D). It is of interest that the whole-cell K_ATP_ conductance is ∼3-fold larger in isolated rat α cells (Olsen et al., 2005) than that we observe in intact mouse islets. This may account for the paradoxical glucose-induced stimulation of glucagon secretion observed in single α cells.

Recently it was reported that the impaired counterregulation of glucagon secretion in diabetic rats could be restored by a somatostatin receptor antagonist (Yue et al., 2012), suggesting that somatostatin signaling is enhanced in diabetic islets. The effects of somatostatin on α cells include activation of K^+^-channels (GIRK) (Kailey et al., 2013). The resulting increase in GIRK-channel activity could be envisaged to have the same effect on glucagon secretion as a low concentration of diazoxide or weak expression of mutant K_ATP_-channels.

Unexpectedly, glucagon secretion is close to normal in α-V59M islets, although α cell electrical activity is strongly affected (Figures 6C and 6D). This is consistent with the mild metabolic phenotype of α-V59M mice (Figures S4A and S4B) and suggests that glucagon secretion is upregulated in the 20% of α cells that remain active. However, the response to adrenaline was reduced by >70% (Figure 6E) in α-V59M islets, a finding that may explain the impaired insulin tolerance of α-V59M mice (Figure S4C). The stimulatory effect of adrenaline on glucagon secretion involves electrical activity and requires influx of extracellular Ca^2+^ (De Marinis et al., 2010). Thus, it seems likely that in α cells expressing mutant K_ATP_-channels, which are electrically silent, adrenaline will be without stimulatory effect. Collectively, these findings argue that the subset of α cells in α-V59M islets that remain electrically active operate close to their maximum secretory capacity already at 1 mM glucose, accounting for the smallness of the adrenaline effect. This is reminiscent of reports of near-normal glucagon secretion in islets after almost total (98%) ablation of α cells by diphtheria toxin (Thorel et al., 2011). If this hypothesis is correct, then glucagon secretion evoked by experimental paradigms that bypass action potential firing should be enhanced in α-V59M islets. Indeed, high-[K^+^]_o_ depolarization (which stimulates secretion in both electrically active and electrically silent α cells) produces a much larger stimulation of glucagon secretion in α-V59M than in control islets (Figure 6F).

It may seem surprising that glucose remains capable of inhibiting glucagon secretion in α-V59M islets despite no detectable glucose-induced reduction of K_ATP_-channel activity. However, for the reasons we outlined above, glucagon secretion will principally reflect the activity of the small subset of cells in which K_ATP_-channel activity is normal. By contrast, measurements of K_ATP_-channel activity will be dominated by α cells expressing high levels of mutant K_ATP_ channels, which respond poorly to glucose. Importantly, in some α cells, expression of mutant K_ATP_-channels is low and total K_ATP_-channel activity only marginally increased, just sufficient to suppress electrical activity at 1 mM glucose. However, when glucose is elevated and K_ATP_-channel activity is reduced, these α cells undergo depolarization and start firing action potential with resultant stimulation of glucagon secretion. This opposes the glucose-induced inhibition in other cells and explains the reduced glucose-induced inhibition of glucagon secretion in α-V59M mutant islets.

In general, the subtle impact of targeting the Kir6.2-V59M mutation to the α cell on glucagon secretion suggests that enhanced α cell K_ATP_-channel activity is unlikely to affect glucagon secretion in patients with neonatal diabetes due to gain-of-function K_ATP_-channel mutations.

### Implications for Type 2 Diabetes

In T2D, glucagon secretion in vivo is often stimulated rather than inhibited during hyperglycemia (Dunning et al., 2005). We observed a similar abnormality in 50% of islet preparations from T2D organ donors (Figure 7A); the remaining preparations exhibited normal glucose regulation. Thus, T2D islets are heterogeneous with respect to the dysregulation of glucagon secretion, in agreement with a recent report (Li et al., 2013). Intriguingly, the inverted response to glucose seen in some T2D islet preparations can be mimicked in ND islets by a small increase (0.5%) in K_ATP_-channel activity produced by diazoxide (Figure 7B). Similarly, metabolic inhibition, which activates K_ATP_-channels, affects glucagon secretion in the same way as T2D (Figure 7D). This suggests that T2D may be associated with increased K_ATP_-channel activity, possibly as a consequence of impaired islet metabolism (Doliba et al., 2012). It is notable that glucagon secretion in the presence of diazoxide or oligomycin shows not only an inverted glucose response but also reduced secretion at 1 mM glucose. This is reminiscent of the impaired counterregulation that is a feature of longstanding T2D, raising the interesting possibility that T2D may involve a progressive and time-dependent increase in K^+^-channel activity and deterioration of the metabolic regulation of glucagon secretion. It is therefore of interest that a *low* concentration of the K_ATP_-channel blocker tolbutamide restores normal glucose regulation of glucagon secretion in metabolically compromised islets (Figure 7D) and improves it in islets from donors with T2D (Figures 7E and 7F). Possibly, low-dose sulfonylurea (much less than required to stimulate insulin secretion) may be a useful addition to insulin therapy. Finally, our data suggest that a single cellular disturbance (impaired glucose metabolism and ATP production), via increased K_ATP_-channel activity, may explain the trio of hormone secretion defects associated with T2D: impaired glucose-induced insulin secretion, inverted glucose regulation of glucagon secretion, and defective counterregulation.

## Experimental Procedures

All experiments were conducted in accordance with the UK Animals Scientific Procedures Act (1986) and University of Oxford ethical guidelines.

### Media

Media used are specified in Table S1.

### Animals and Generation of α-V59M Mice

Most experiments were performed on islets isolated from NMRI mice obtained from a commercial supplier. Mice expressing Kir6.2-V59M in α cells (α-V59M mice) were generated using a Cre-lox approach (Clark et al., 2010).

### Human Islets

Human islets (obtained with ethical approval and clinical consent) were isolated from pancreases of 46 nondiabetic donors and 10 donors with T2D.

### Genotyping of Human Islet DNA

The rs5219 variant was genotyped using an allelic discrimination assay-by-design method on an ABI 7900 analyzer (Applied Biosystems).

### Hormone Release Measurements

Measurements of insulin and glucagon secretion were performed using the in situ pancreas perfusion or static incubations of isolated islets.

### Electrophysiology

All electrophysiological measurements were performed at +34°C on α cells within intact islets (from NMRI or α-V59M and control mice).

For the membrane potential and whole-cell K_ATP_-current recordings (Figures 2A–2C), the perforated patch technique was employed as reported previously (De Marinis et al., 2010). The pipette solution consisted of IC1, and the bath contained EC2.

Exocytosis was measured as increases in membrane capacitance in α cells in intact islets as described previously (Göpel et al., 2004) using pipette medium IC2 and extracellular medium EC3. The impact of glucose on exocytosis was tested using the perforated patch technique using pipette medium IC3.

Voltage-dependent inactivation of the Na^+^-current was evaluated using the standard whole-cell technique and a two-pulse protocol using intra- and extracellular media IC4 and EC4, respectively.

### ATP Imaging

The ATP/ADP sensor Perceval (Berg et al., 2009) was used as previously described (Tarasov et al., 2012).

### Identification of α Cells

The identity of the α cells was established either by (1) immunocytochemistry following injection of the cell with biocytin (0.5 mg/ml) via the recording electrode (Figures S2C and S2D) or (2), in the case of α-V59M and control α cells, by tdRFP fluorescence. In perforated patch measurements of electrical activity and K_ATP_-channel activity in NMRI islets (Figures 2–4), it was not always possible to identify the cell by immunocytochemistry because the cell detached when retracting the recording electrode. In these cases, α cells were identified by their spontaneous action potential firing at 1 mM glucose (Göpel et al., 2000a).

### Statistical Analysis

Details on the analysis of the electrophysiological data are given in the Supplemental Information. Data are presented as man values ± SEM of the indicated number of experiments (n). Error bars in figures represent SEM. Statistical significances were, unless otherwise indicated, evaluated using Student’s t test.

## Supplemental Information

Document S1. Supplemental Experimental Procedures, Figures S1–S6, and Table S1
