# Supplementary material for: Role of KATP Channels in Glucose-Regulated Glucagon Secretion and Impaired Counterregulation in Type 2 Diabetes
Source: Cell Metab. 2013 Dec 3;18(6):871–82. doi: 10.1016/j.cmet.2013.10.014 (PMC3851686; doi:10.1016/j.cmet.2013.10.014)
Supplement: Document S1. Supplemental Experimental Procedures, Figures S1–S6, and Table S1 [file mmc1.pdf]

Cell Metabolism, Volume 18

## Supplemental Information

# Role of $K_{ATP}$ Channels in Glucose-Regulated Glucagon Secretion and Impaired Counterregulation in Type 2 Diabetes

Quan Zhang, Reshma Ramracheya, Carolina Lahmann, Andrei Tarasov, Martin Bengtsson, Orit Braha, Matthias Braun, Melissa Brereton, Stephan Collins, Juris Galvanovskis, Alejandro Gonzalez, Lukas N. Groschner, Nils J.G. Rorsman, Albert Salehi, Mary E. Travers, Jonathan N. Walker, Anna L. Gloyn, Fiona Gribble, Paul R.V. Johnson, Frank Reimann, Frances M. Ashcroft, and Patrik Rorsman

## 1. Supplemental Information. Methods

### Generation of α-V59M mice

Mice expressing Kir6.2-V59M in α-cells (α-V59M mice) were generated using a Cre-lox approach. The *KCNJ11* gene encoding a mutant Kir6.2-V59M subunit, preceded by a loxP-flanked STOP sequence and followed by an FRT-flanked internal ribosome entry site and a GFP cassette was targeted to the ROSA26 locus to ensure that a single copy of the mutant gene was expressed from a known location (Girard et al., 2009). The endogenous ROSA promoter was used to prevent excess gene expression. To generate mice that express the transgene specifically in α-cells, ROSA26StopKir6.2-V59M$^{lox/+}$ mice (ROSA mice) were crossed with mice expressing Cre recombinase under the control of the pro-glucagon promoter (Parker et al., 2012). This generated mice (α-V59M mice) in which expression of Cre recombinase in glucagon-producing α-cells leads to deletion of the STOP cassette and thus to expression of the mutant gene encoding Kir6.2-V59M. Proglucagon-Cre littermates were used as controls. All mice carrying the proglucagon-Cre gene were positive for tandem-dimer red fluorescent protein (tdRFP), so that α-cells in which Cre recombinase was activated (and thus expressed the Kir6.2-V59M gene) fluoresced red (PPG-RFP). It was ascertained that cells expressing tdRFP are also positive for glucagon (Figure S1). Genotypes were identified by PCR using genomic DNA isolated from ear biopsies, as previously described (Clark et al., 2010). All experiments were carried out blinded to genotype.

## Hormone release measurements

Batches of 10–20 size-matched islets were preincubated in 0.3ml of a modified Krebs-Ringer buffer (EC1) containing 1 glucose and 2mg/ml BSA for 1h at 37°C, followed by a 1h test incubation in 0.3ml of the same medium supplemented with glucose, receptor antagonists, metabolic inhibitors and ion channel activators/blockers as indicated.

Dynamic measurements of insulin secretion were performed using *in situ* pancreatic perfusion. Briefly, the aorta was cannulated by ligating above the coeliac artery and below the superior mesenteric artery, and the pancreas was perfused with EC1 at a rate of ~0.45ml/min using an Ismatec Reglo Digital MS2/12 peristaltic pump. The perfusate was maintained at +37$^{o}$C with a Warner Instruments temperature control unit TC-32 4B in conjunction with a tube heater (Warner Instruments P/N 64-0102) and a Harvard Apparatus heated rodent operating table. The effluent was collected by cannulating the portal vein and using a Teledyne ISCO Foxy R1 fraction collector. The pancreas was first perfused for 20min with 6mM glucose before commencing the experiment to establish the basal rate of secretion.

## Electrophysiology

The electrophysiological measurements were performed on $\alpha$-cells within intact islets (from NMRI, $\alpha$-V59M or control mice), using an EPC-10 patch-clamp amplifier (HEKA Electronics, Lambrecht/Pfalz, Germany) and Pulse (version 8.80) software. All electrophysiological experiments were performed at +34$^{o}$C.

For the membrane potential and conductance recordings (Figure 2A-C), the perforated patch technique was employed. The pipette solution consisted of IC1 and the bath contained EC2. Changes in whole-cell resting conductance were measured in α-cells within intact islets in response to ±10mV pulses from the holding potential (-70mV). The membrane conductance (G) was calculated by dividing the current response ($\Delta I$) with the voltage change ($\Delta V$).

For current injection experiments, spontaneous action potential firing was first recorded in 1mM glucose, and then suppressed by injecting negative current (a few pA) via the recording electrode to hold the membrane potential at ~-80mV. Action potentials were then elicited by application of progressively larger depolarizing currents. The peak voltage was then measured for each potential once the membrane had stabilized at the new membrane potential (ignoring action potentials occurring during the depolarization to the new plateau).

Exocytosis was measured as increases in membrane capacitance in α-cells in intact islets from NMRI mice as described previously (Gopel et al., 2004). When using the standard whole-cell technique (Figure 5A,C,D), intra- and extracellular media IC2 and EC2 were used. The impact of glucose on exocytosis was tested using the perforated patch technique, pipettes were filled with IC1 and the bath perfused with EC2 medium plus glucose as indicated. The effect of action potential height on the P/Q-type $Ca^{2+}$-current was evaluated in the standard whole-cell measurements by first averaging >20 action potentials (at either 1 or 6mM glucose) in a representative experiment. The resulting voltage waveforms were then used as the voltage-clamp commands.

Voltage-dependent inactivation of the $Na^+$-current was evaluated by a standard two-pulse protocol applied at 1Hz in which a 200ms conditioning pulse to membrane potentials between -100 and -10mV preceded at test pulse to 0mV. In these experiments, the pipettes were filled with IC3 and bath perfused with EC4 in which $Co^{2+}$ replaced $Ca^{2+}$ to block inward $Ca^{2+}$-currents.

### $[Ca^{2+}]_i$ imaging

Confocal $[Ca^{2+}]_i$ imaging experiments on α-V59M islets were conducted essentially as previously reported (Girard et al., 2009). Islets were superfused with EC1 medium plus glucose or tolbutamide as indicated, at a flow rate of 1ml/min and 37$^oC$.

### Quantitative PCR

Methods for preparation of α-cell cell fractions and quantitative PCR are as detailed previously (De Marinis et al., 2010)) for details. Primer sequences are available on request.

### ATP imaging

The adenovirus encoding the ATP/ADP sensor Perceval (Berg et al., 2009) was generated as described (Tarasov et al., 2012). Intact islets isolated from PPG-tdRFP mice (see above) were infected with the adenovirus at ~$10^6$pfu/islet. Imaging experiments were performed on a Zeiss 510 META upright confocal microscope, using a 40x (n.a. 0.8) objective. Islets were superfused with EC1 solution with glucose and FCCP being added as indicated (Figure 1B). Excitation/emission wavelengths were (nm) 490/535 (Perceval) and 567/610

(tdRFP). Images were acquired at a frequency of 0.05-0.1Hz. Imaging data was background-subtracted, analyzed and presented as the increase in $F/F_0$ using ImageJ and Igor Pro software (Wavemetrics). We considered the possibility that a potential interference between Perceval and tdRFP might affect $[ATP]/[ADP]_i$ measurements in tdRFP-positive α-cells. We therefore monitored $[ATP]/[ADP]_i$ in α- and β-cells identified by the occurrence of spontaneous $[Ca^{2+}]_i$ oscillations at low (1 mM, α-cells ) and high (20mM, β-cells) glucose, respectively (cf. Figure S1C-D but using fura red instead of fluo-4). The responses in α-cells identified by spontaneous $[Ca^{2+}]_i$ oscillations at 1mM glucose had similar kinetics and magnitude to those observed in tdRFP-positive cells.

## Identification of α-cells

In all voltage-clamp experiments, the identity of the α-cells was established either by (i) immunocytochemistry following injection of the cell with biocytin (0.5mg/ml) via the recording electrode (Zhang et al., 2007); or (ii) in the case of α-V59M and control α-cells, by RFP fluorescence. It was confirmed that tdRFP fluorescence was confined to glucagon-positive cells and not in cells with insulin or somatostatin immunoreactivity. The same observations were made in freshly and following 48h of tissue culture. In confocal $[Ca^{2+}]_i$ imaging experiments, 94±2% (n=8 mice) of the tdRFP-positive cells generated spontaneous $[Ca^{2+}]_i$ oscillations when exposed to 1 mM glucose (as expected for α-cells but not β- or δ-cells). In perforated patch measurements of electrical activity and $K_{ATP}$-channel activity in NMRI islets (Figures 2-4), it was not always possible to subsequently identify the

cell by immunocytochemistry because the cell detached from the islet when retracting the recoding electrode (but see Figure S1A-B). In these cases, α-cells were identified based on their spontaneous action potential firing at 1mM glucose; insulin-secreting β-cells and somatostatin-secreting δ-cells are electrically silent at low glucose (Gopel et al., 2000).

## Glucose and insulin tolerance tests

12-week-old α-V59M mice and control littermates were fasted overnight (15-16 hours) and a fasted blood sample taken from a tail vein under local anaesthesia (5% EMLA cream, AstraZeneca). For IPGTT, mice were injected intraperitoneally with a glucose load of 2g/kg body weight (20% glucose in 0.9% NaCl). For OGTT, mice were given a glucose load of 2g/kg body weight by oral gavage (10% glucose in 0.9% NaCl). Blood samples were obtained 30, 60 and 120min after glucose administration. Plasma glucose was measured using a Freestyle Lite handheld glucose meter (Abbott).

For the insulin tolerance tests, experimental procedures were the same except that free-fed mice were used and insulin was administered at 1U/kg of body weight (0.1U/mL insulin in 0.9% NaCl). Blood samples were obtained 15, 30, 45, 60, and 90 minutes after insulin injection.

## Data analysis and statistics

Action potential peak voltage, the most negative interspike membrane potential and firing frequency were analyzed using the event detection/threshold search routine of Clampfit 9 (Molecular Devices, Sunnyvale, CA). For the experiment in

Figure 2, 50-900 action potentials were averaged during a 1-9min period around the indicated time points and the values thus obtained were used to calculate the averages for the experimental series. In the current-injection experiments shown in Figure 4A-C, the peak voltage and the most negative preceding interspike membrane potential were measured for each action potential during different current pulses. The data points thus obtained were fit to a Boltzmann relationship to derive the maximum and minimum peak voltages, the midpoint and the slope factor for the individual experiment. For display (Figure 4C), the responses were sorted according to the inter-spike membrane potential and binned (bin width 5mV; i.e. -65 to -60mV; -59.9 to -55mV etc.) and the mean inter-spike membrane potential and peak voltage calculated for the action potentials within each bin.

All data are given as mean ± standard error of the mean (SEM) of the indicated number of experiments (cells for electrophysiology, replicates or mice for other experiments; n). Error bars in Figures indicate SEM. Statistical significances were, unless otherwise indicated, evaluated using Student's *t*-test.

## References

Berg, J., Hung, Y.P., and Yellen, G. (2009). A genetically encoded fluorescent reporter of ATP:ADP ratio. Nat Methods *6*, 161-166.

Clark, R.H., McTaggart, J.S., Webster, R., Mannikko, R., Iberl, M., Sim, X.L., Rorsman, P., Glitsch, M., Beeson, D., and Ashcroft, F.M. (2010). Muscle dysfunction caused by a KATP channel mutation in neonatal diabetes is neuronal in origin. Science *329*, 458-461.

De Marinis, Y.Z., Salehi, A., Ward, C.E., Zhang, Q., Abdulkader, F., Bengtsson, M., Braha, O., Braun, M., Ramracheya, R., Amisten, S., *et al.* (2010). GLP-1 inhibits and adrenaline stimulates glucagon release by differential modulation of N- and L-type Ca2+ channel-dependent exocytosis. Cell Metab *11*, 543-553.

Girard, C.A., Wunderlich, F.T., Shimomura, K., Collins, S., Kaizik, S., Proks, P., Abdulkader, F., Clark, A., Ball, V., Zubcevic, L., *et al.* (2009). Expression of an activating mutation in the gene encoding the KATP channel subunit Kir6.2 in mouse pancreatic beta cells recapitulates neonatal diabetes. J Clin Invest *119*, 80-90.

Gopel, S., Zhang, Q., Eliasson, L., Ma, X.S., Galvanovskis, J., Kanno, T., Salehi, A., and Rorsman, P. (2004). Capacitance measurements of exocytosis in mouse pancreatic alpha-, beta- and delta-cells within intact islets of Langerhans. The Journal of physiology *556*, 711-726.

Gopel, S.O., Kanno, T., Barg, S., and Rorsman, P. (2000). Patch-clamp characterisation of somatostatin-secreting -cells in intact mouse pancreatic islets. The Journal of physiology *528*, 497-507.

Le Marchand, S.J., and Piston, D.W. (2010). Glucose suppression of glucagon secretion: metabolic and calcium responses from alpha-cells in intact mouse pancreatic islets. The Journal of biological chemistry *285*, 14389-14398.

MacDonald, P.E., De Marinis, Y.Z., Ramracheya, R., Salehi, A., Ma, X., Johnson, P.R., Cox, R., Eliasson, L., and Rorsman, P. (2007). A K ATP channel-dependent pathway within alpha cells regulates glucagon release from both rodent and human islets of Langerhans. PLoS Biol *5*, e143.

Parker, H.E., Adriaenssens, A., Rogers, G., Richards, P., Koepsell, H., Reimann, F., and Gribble, F.M. (2012). Predominant role of active versus facilitative glucose transport for glucagon-like peptide-1 secretion. Diabetologia *55*, 2445-2455.

Quoix, N., Cheng-Xue, R., Guiot, Y., Herrera, P.L., Henquin, J.C., and Gilon, P. (2007). The GluCre-ROSA26EYFP mouse: a new model for easy identification of living pancreatic alpha-cells. FEBS letters *581*, 4235-4240.

Tarasov, A.I., Semplici, F., Ravier, M.A., Bellomo, E.A., Pullen, T.J., Gilon, P., Sekler, I., Rizzuto, R., and Rutter, G.A. (2012). The mitochondrial Ca2+ uniporter MCU is essential for glucose-induced ATP increases in pancreatic beta-cells. PloS one *7*, e39722.

Zhang, Q., Bengtsson, M., Partridge, C., Salehi, A., Braun, M., Cox, R., Eliasson, L., Johnson, P.R., Renstrom, E., Schneider, T., *et al.* (2007). R-type Ca(2+)-channel-evoked CICR regulates glucose-induced somatostatin secretion. Nat Cell Biol *9*, 453-460.

Zhang, Q., Galvanovskis, J., Abdulkader, F., Partridge, C.J., Gopel, S.O., Eliasson, L., and Rorsman, P. (2008). Cell coupling in mouse pancreatic beta-cells measured in intact islets of Langerhans. Philos Transact A Math Phys Eng Sci *366*, 3503-3523.

## 2. Supplemental information. Supplemental Data.

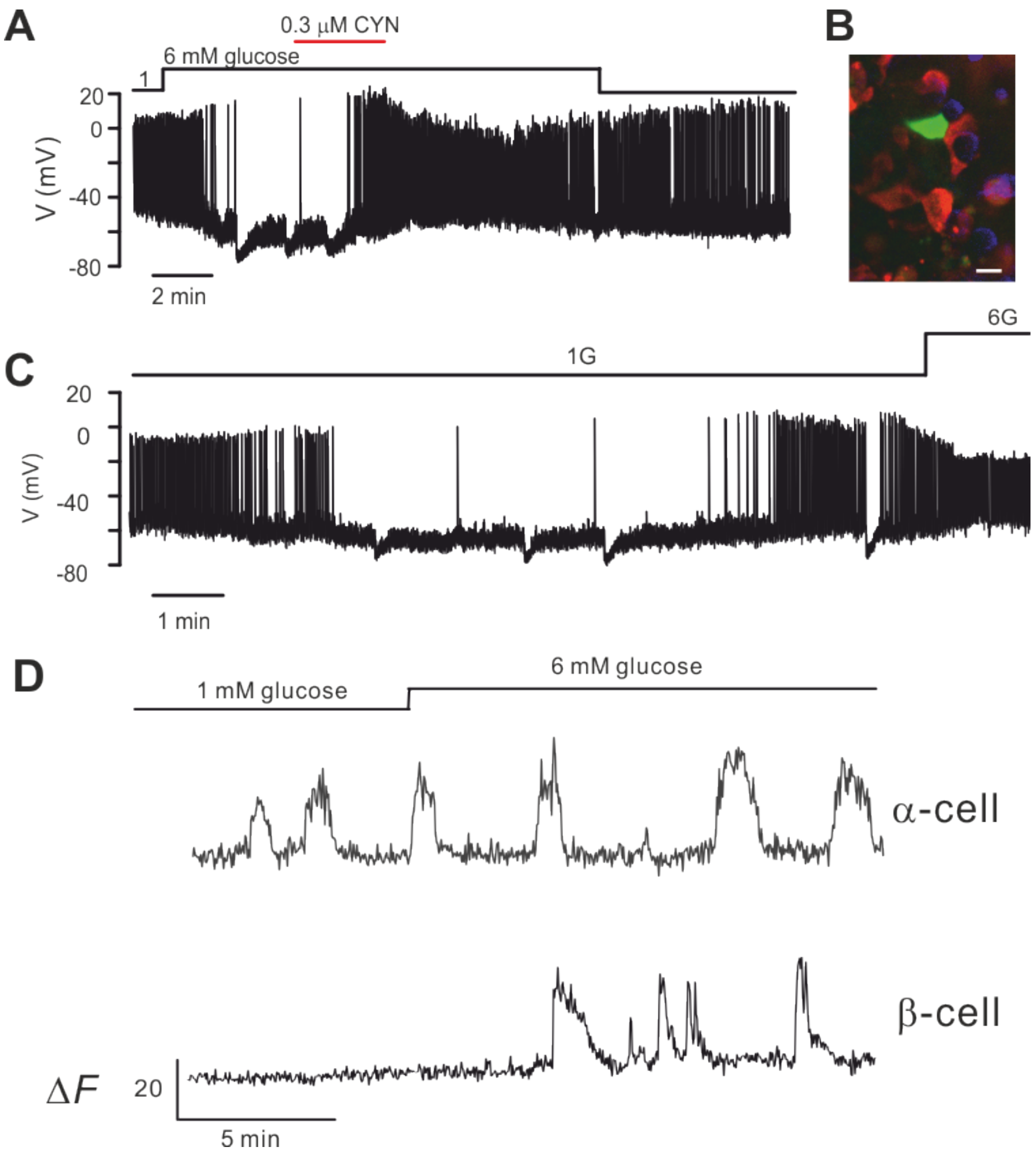


**Figure S1. Transient repolarization in some α-cells and weak effects of $[Ca^{2+}]_i$ in α-cells (related to Figure 2).**

(A) Electrical activity in a mouse α-cell in an intact mouse islet at 1mM and 6mM glucose. Note the repolarization following the addition of glucose, that action potential firing resumes following addition of the SSTR2 antagonist CYN154806 (0.3μM), the maintained action potential firing in the continued presence of 6mM glucose following washout of CYN154806, and the membrane repolarization and increase in action potential height when glucose was subsequently lowered to 1mM

(B) Immunocytochemistry showing that the α-cell (glucagon+) used for the membrane potential recording in A (highlighted in green by the presence of biocytin) was juxtaposed to a somatostatin-producing δ-cell (red). Blue indicates glucagon immunoreactivity (α-cells) Scale bar: 10μm.

(C) As in (A) but showing the spontaneous ~5min repolarization followed by resumption of electrical activity in the continued presence of 1mM glucose, and the effect of subsequently applying 6mM glucose.

(D) $[Ca^{2+}]_i$ measured in α-cells (top, grey trace) and β-cells (lower, black trace) in intact pancreatic islets at 1mM and 6mM glucose as indicated. Note glucose did not affect $[Ca^{2+}]_i$ in α-cells during the time required to initiate oscillations in β-cells. Traces are representative of recordings from a total of 6 out of 111 β-cells and 34 out of 46 α-cells (from 9 different islets). At 6mM glucose, few β-cells responded to glucose; however, at 20mM glucose all 111 β-cells responded with an increase in $[Ca^{2+}]_i$. The fact that glucagon secretion is modulated by glucose without major changes in $[Ca^{2+}]_i$ has been taken to indicate that glucose regulates insulin secretion downstream of membrane depolarization (Le Marchand and Piston, 2010). However, global measurements do not necessarily reflect $[Ca^{2+}]_i$ in the vicinity of the release sites, which will be much higher if the release sites and $Ca^{2+}$-channels are colocalized. Importantly, glucagon release is coupled to $Ca^{2+}$entry through P/Q type $Ca^{2+}$ channels, which was reduced at high glucose. $Ca^{2+}$ influx through L-type $Ca^{2+}$ channels is, however, increased in parallel with the increased spike frequency. This might explain the apparent dichotomy between $[Ca^{2+}]_i$ and glucagon release.

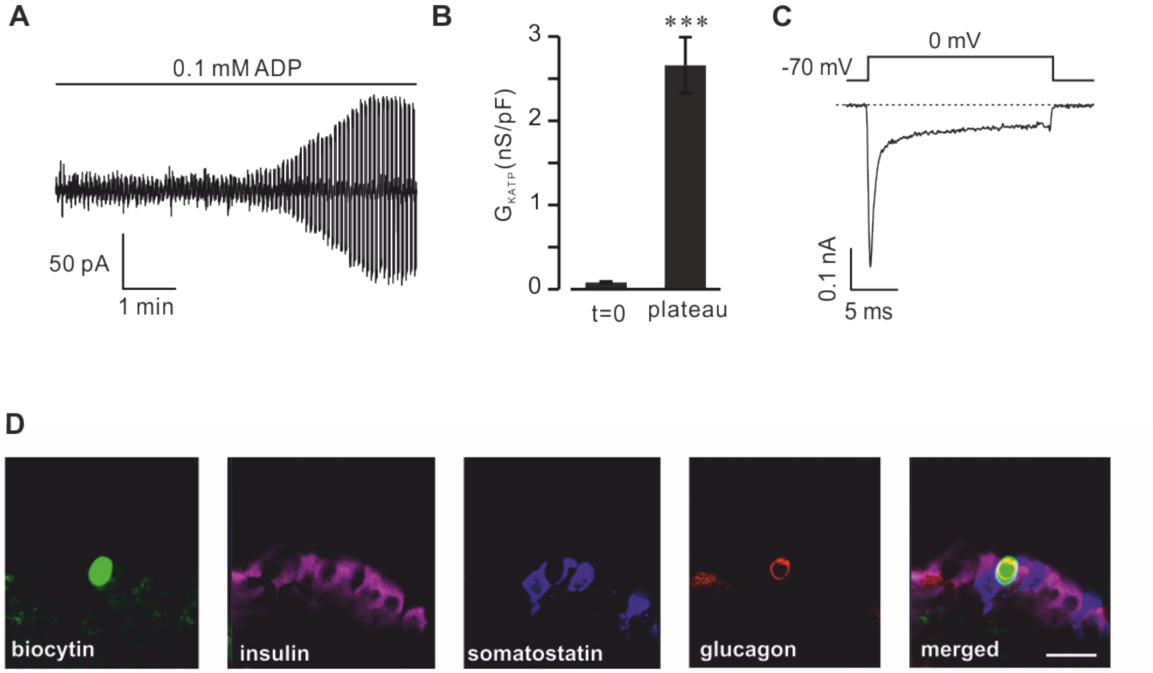


**Figure S2. Activation of $K_{ATP}$-channels following intracellular application of ADP and identification of $\alpha$-cells using biocytin-fillied electrodes (related to Figures 2-3).**

(A) Time-dependent increase in membrane conductance in wild-type $\alpha$-cells in intact islets following the establishment of the whole-cell configuration and intracellular application of ADP (0.1mM) and washout of ATP. The cell was held at -70 mV and $K_{ATP}$-channel activity monitored by application of ±10 mV voltage-clamp depolarizations. The progressive increase in the current deflection indicates the activation of the $K_{ATP}$-channels.

(B) Membrane conductance measured immediately after establishment of the whole-cell configuration (t=0) and when a new (larger) steady-state plateau value had been attained. ***$p<0.001$ vs. initial value. Data are presented as mean values ± SEM.

(C) Voltage-clamp current recorded from a cell in an intact islet using a Cs-filled electrode solution (IC2) supplemented with biocytin during a depolarization from -70mV to 0mV. Note biphasic current. The initial spiky and sustained components represent the TTX-sensitive $Na^+$-current and the voltage-gated $Ca^{2+}$-current (P/Q and L-type), respectively.

(D) Immunocytochemistry showing a cell loaded with biocytin (green) via the electrode used for the patch-clamp measurements also positive for glucagon (red) and the merged signal (yellow, far right). Also shown are surrounding $\beta$-(insulin) and $\delta$-cells (somatostatin). Scale bar: 20$\mu$m.

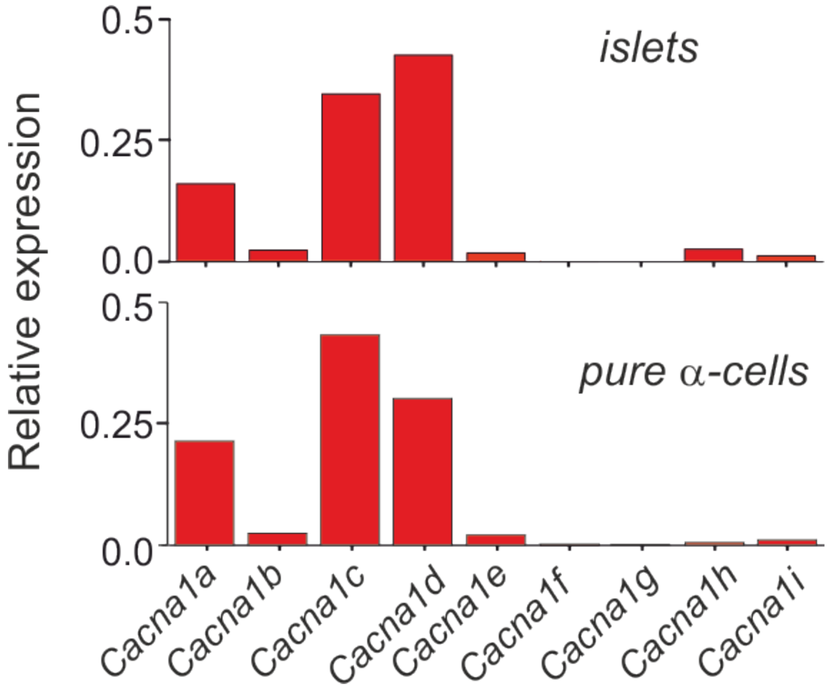


**Figure S3. $Ca^{2+}$-channels expressed by α-cells (related to Figure 5)**

Relative expression of different $Ca^{2+}$-channel subunits. Quantitative PCR of mRNA obtained from C57BL/6 mouse islets (top) and pure α-cell fractions (below). Data are normalized to the sum of all *Cacna* transcripts. Note the high expression of *Cacna1a* (P/Q-type), *Cacna1c* (L-type, $\alpha_{1C}$) and *Cacna1d* (L-type, $\alpha_{1D}$). Other subunits (including *Cacna1b*; N-type) are expressed at much lower levels.

We have previously shown that the dihydropyridine-resistant (non-L-type) $Ca^{2+}$-current in α-cells is sensitive to ω–conotoxin (MacDonald et al., 2007), suggesting it might be carried by N-type channels. However, the above transcript analysis suggests that ω-conotoxin-sensitive *Cacna1b* $Ca^{2+}$-channels (N-type ) are expressed at very low levels in mouse islets, whereas *Cacna1a* ω-agatoxin-sensitive P/Q-type $Ca^{2+}$-channels are expressed at 10-fold higher levels. As ω-conotoxin exerted little or no additional inhibitory effect on whole-cell $Ca^{2+}$-currents in α-cells already exposed to ω-agatoxin, the effects of ω-conotoxin are likely to reflect non-specific blockade of P/Q-type $Ca^{2+}$-channels.

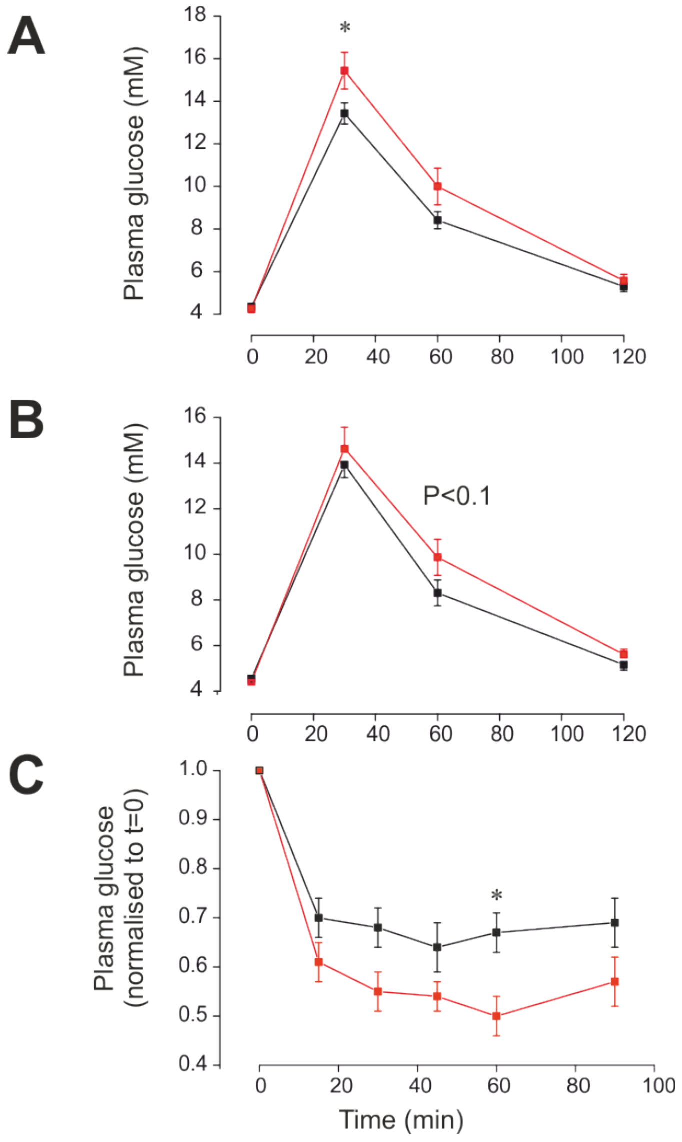


**Figure S4. Impaired glucose and insulin tolerance in α-V59M mice (related to Figure 6)**

(A) Plasma glucose concentrations measured following an intraperitoneal glucose challenge (at t=0) in control (black, n=21) and α-V59M (red, n=22) mice.*p<0.05.

(B) Plasma glucose concentrations measured following an oral glucose challenge in control (black, n=21) and α-V59M (red, n=22) mice.

(C) Insulin tolerance test. Plasma glucose concentrations measured following injection of insulin (at t=0) in control (black, n=13) and α-V59M (red, n=20) mice. Data have been normalized to basal plasma glucose (6.6±0.2mM in α-V59M mice vs. 6.1±0.3mM in control mice; not statistically different). *p<0.05 vs. corresponding values in control mice. Data are presented as mean values ± SEM.

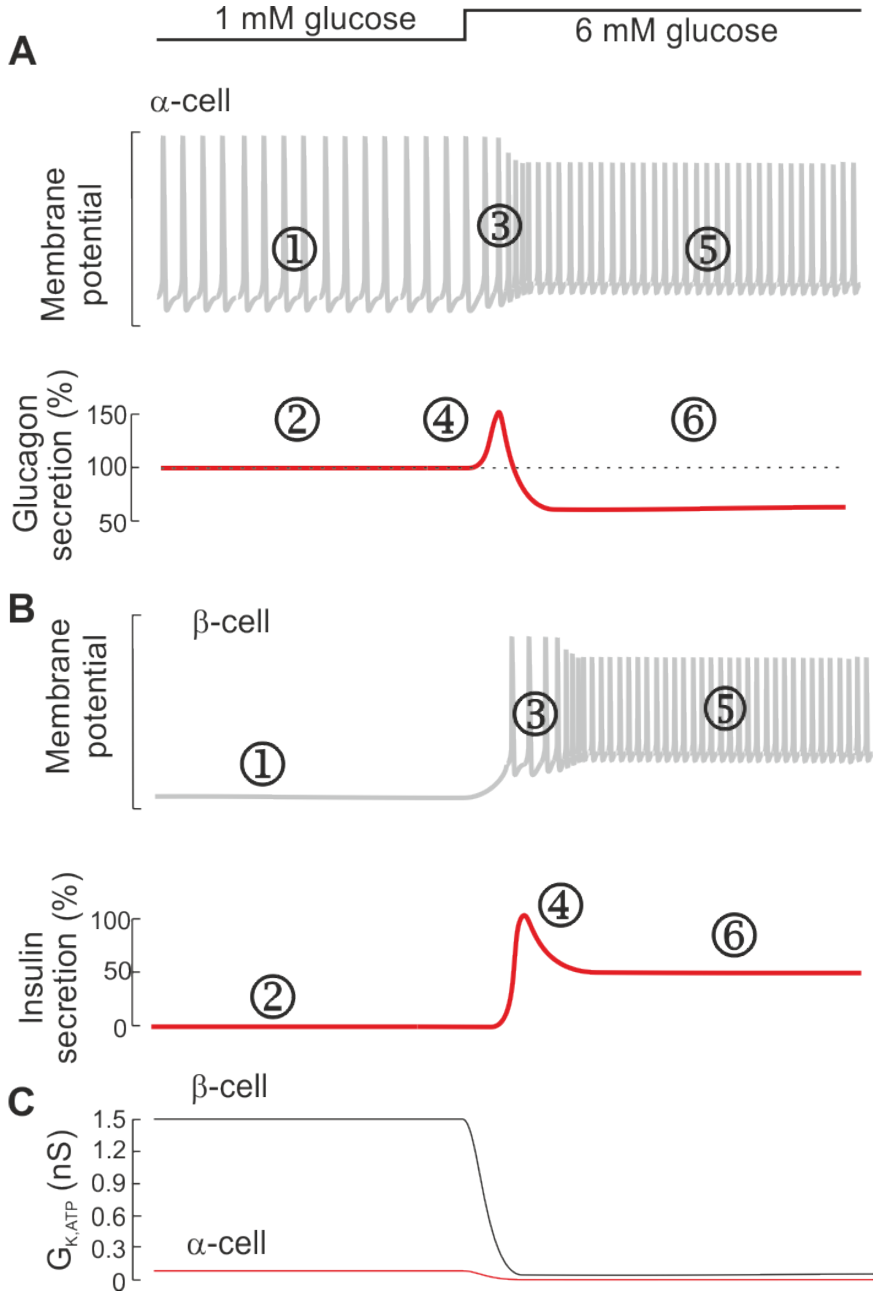


**Figure S5. Dual effects of K$^+$-channel closure in α- and β-cells (related to Figure 7).**

Schematic explaining the effects of glucose on electrical activity and hormone secretion in α-cells and β-cells.

(A) The α-cell is electrically *active* at 1mM glucose (①) which stimulates glucagon secretion ②. Increasing glucose to 6mM increases spike frequency (③) and transiently stimulates glucagon secretion (④) (cf. (Quoix et al., 2007)). However, the stimulation of action potential firing is insufficient to fully compensate for the reduction of exocytosis resulting from the reduced spike height (due to inactivation of ion channels involved in action potential firing; ⑤). Thus the net effect is suppression of glucagon secretion (⑥).

(B) The β-cell is electrically *silent* at 1mM glucose (①) and thus insulin secretion is minimal (②). Glucose triggers electrical activity (③) and thereby evokes insulin secretion (④). The reduction of spike height may slightly reduce stimulation (⑤) but the net effect is a strong and persistent stimulation of insulin secretion (⑥).

(C) Glucose-dependent changes in $K_{ATP}$-channel activity in α- (red) and β-cells (black). $K_{ATP}$-channel activity is very low in α-cells compared to β-cells: at 1mM glucose it is comparable (or lower) to that found in β-cells exposed to 6mM glucose. Values for β-cells taken from (Zhang et al., 2008) and for α-cells from this paper.

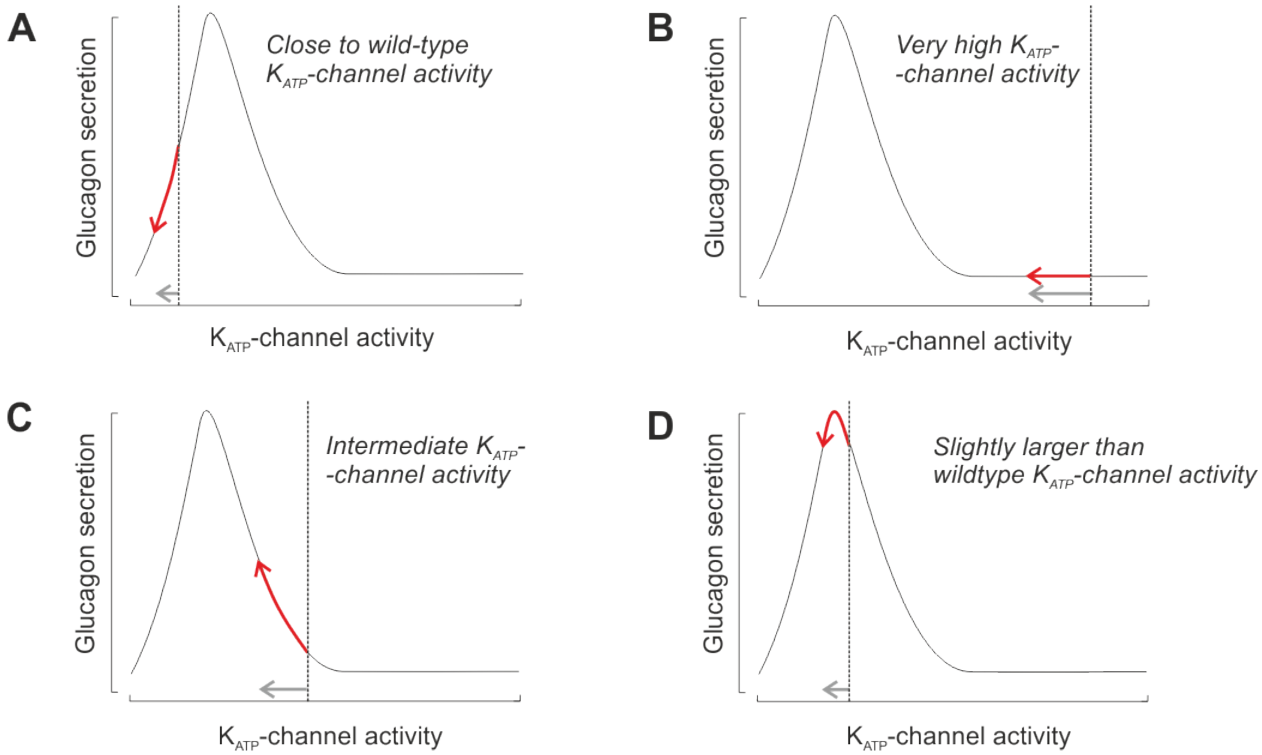


**Figure S6. Effects of glucose on glucagon secretion in islets with variable increase in $K_{ATP}$-channel activity (related to Figure 7)**

(A) Effects of glucose-induced closure of $K_{ATP}$-channels on glucagon secretion in $\alpha$-V59M islets expressing exclusively wild-type $K_{ATP}$-channels. Closure of $K_{ATP}$-channels (grey arrow) leads to inhibition of glucagon secretion (red arrow), analogous to what is seen in control (and wild-type) cells.

(B) As in (A) but showing a cell expressing many V59M $K_{ATP}$-channels. The resting conductance in such cells is so high that glucose-induced closure of $K_{ATP}$-channels will not affect glucagon secretion, which will be low at both 1mM and 6mM glucose (analogous to what is seen for wild-type $\alpha$-cells in the presence of a high concentration of diazoxide).

(C) As in (B) but expressing fewer V59M $K_{ATP}$-channels. In such cells, glucagon secretion will be low at 1mM glucose and elevation to 6mM glucose may reduce $K_{ATP}$-channel activity sufficiently to stimulate glucagon secretion (i.e. these cells have an inverted glucose response, similar to the response seen for wild-type $\alpha$-cells in the presence of a low concentration of diazoxide).

(D) As in (C) but expressing even fewer $K_{ATP}$-channels (but more than in wild-type cells). In such cells, glucagon secretion at 1mM glucose might be higher than in wild-type cells. Glucose will reduce $K_{ATP}$-channel activity but because of the bell-shaped relationship between $K_{ATP}$-channel activity and glucagon secretion, this may not produce inhibition of glucagon secretion.

In A-D, the grey arrows indicate the reduction of $K_{ATP}$-channel activity and the red arrows the change in glucagon secretion. The dashed vertical lines indicate the resting conductance at 1mM glucose.

**3. Supplemental Information. Table.**

## Table S1. Composition of media (Related to Figures 1-7)

The following intra- and extracellular media were used

| Composition of intracellular (IC) pipette-filling media | | | |
|---|---|---|---|
| IC1 (mM) | IC2 (mM) | IC3 (mM) | |
| 76 $K_2SO_4$ | 125 Cs-glutamate | 125 CsCl | |
| 10 NaCl | 10 CsCl | 10 EGTA | |
| 10 KCl | 10 NaCl | 30 CsOH | |
| 1 $MgCl_2$ | 1 $MgCl_2$ | 1 $MgCl_2$ | |
| 5 HEPES (pH 7.35 with KOH) | 5 HEPES (pH 7.15 with CsOH) | 3 Mg-ATP | |
| | 0.05-1 EGTA | 5 HEPES (pH 7.15 with CsOH) | |
| | 3 Mg-ATP | | |
| | | | |
| **Composition of extracellular (EC) media** | | | |
| EC1 (mM) | EC2 (mM) | EC3 (mM) | EC4 (mM) |
| 140 NaCl* | 140 NaCl | 118 NaCl | 118 NaCl |
| 3.6 KCl | 3.6 KCl | 20 TEA-Cl | 20 TEA-Cl |
| 0.5 $MgSO_4$ | 0.5 $MgSO_4$ | 5.6 KCl | 5.6 KCl |
| 0.5 $Na_2H_2PO_4$ | 0.5 $Na_2H_2PO_4$ | 1.2 $MgCl_2$ | 1.2 $MgCl_2$ |
| 2 $NaHCO_3$ | 5 $NaHCO_3$ | 5 HEPES (pH 7.4 with NaOH) | 5 HEPES (pH 7.4 with NaOH) |
| 5 HEPES (pH 7.4 with NaOH) | 10 HEPES (pH 7.4 with NaOH) | 2.6 $CaCl_2$ | 2 $CoCl_2$ |
| 1.3 $CaCl_2$ | 1.3 $CaCl_2$ | | |
| *Correspondingly reduced when $[K^+]_o$ was elevated to maintain iso-osmolarity. | | | |
| | | | |
| **Amino acid mixtures (AAM)** – Included in EC1 in Figure 3D as indicated | | | |
| **AAMa** (mM): 0.15 threonine, 0.15 leucine, 0.2 lysine, 0.1 serine, 0.25 glycine, 0.4 alanine, 0.5 glutamine, 0.25 valine<br><br>**AAMb** (mM): 2 alanine, 2 glutamine and 2 arginine | | | |
